# A Field-Based Approach to Determine Soft Tissue Injury Risk in Elite Futsal Using Novel Machine Learning Techniques

**DOI:** 10.3389/fpsyg.2021.610210

**Published:** 2021-02-05

**Authors:** Iñaki Ruiz-Pérez, Alejandro López-Valenciano, Sergio Hernández-Sánchez, José M. Puerta-Callejón, Mark De Ste Croix, Pilar Sainz de Baranda, Francisco Ayala

**Affiliations:** ^1^Department of Sport Sciences, Sports Research Centre, Miguel Hernández University of Elche, Elche, Spain; ^2^Centre for Sport Studies, King Juan Carlos University, Madrid, Spain; ^3^Department of Pathology and Surgery, Physiotherapy Area, Miguel Hernandez University of Elche, Alicante, Spain; ^4^Department of Computer Systems, University of Castilla-La Mancha, Albacete, Spain; ^5^School of Sport and Exercise, University of Gloucestershire, Gloucester, United Kingdom; ^6^Department of Physical Activity and Sport, Faculty of Sports Sciences, University of Murcia, Murcia, Spain; ^7^Ramón y Cajal Postdoctoral Fellowship, Department of Physical Activity and Sport, Faculty of Sports Sciences, University of Murcia, Murcia, Spain

**Keywords:** injury prevention, modeling, screening, decision-making, algorithm, decision tree

## Abstract

Lower extremity non-contact soft tissue (LE-ST) injuries are prevalent in elite futsal. The purpose of this study was to develop robust screening models based on pre-season measures obtained from questionnaires and field-based tests to prospectively predict LE-ST injuries after having applied a range of supervised Machine Learning techniques. One hundred and thirty-nine elite futsal players underwent a pre-season screening evaluation that included individual characteristics; measures related to sleep quality, athlete burnout, psychological characteristics related to sport performance and self-reported perception of chronic ankle instability. A number of neuromuscular performance measures obtained through three field-based tests [isometric hip strength, dynamic postural control (Y-Balance) and lower extremity joints range of motion (ROM-Sport battery)] were also recorded. Injury incidence was monitored over one competitive season. There were 25 LE-ST injuries. Only those groups of measures from two of the field-based tests (ROM-Sport battery and Y-Balance), as independent data sets, were able to build robust models [area under the receiver operating characteristic curve (AUC) score ≥0.7] to identify elite futsal players at risk of sustaining a LE-ST injury. Unlike the measures obtained from the five questionnaires selected, the neuromuscular performance measures did build robust prediction models (AUC score ≥0.7). The inclusion in the same data set of the measures recorded from all the questionnaires and field-based tests did not result in models with significantly higher performance scores. The model generated by the UnderBagging technique with a cost-sensitive SMO as the base classifier and using only four ROM measures reported the best prediction performance scores (AUC = 0.767, true positive rate = 65.9% and true negative rate = 62%). The models developed might help coaches, physical trainers and medical practitioners in the decision-making process for injury prevention in futsal.

## Introduction

Lower extremity non-contact soft tissue (muscle, tendon, and ligament) (LE-ST) injuries are very common events in intermittent team sports such as soccer (López-Valenciano et al., 2019), futsal (Ruiz-Pérez et al., 2020), rugby (Williams et al., 2013), bat (i.e., cricket and softball) and stick (i.e., field hockey and lacrosse) sports (Panagodage Perera et al., 2018). It has been suggested that most of these LE-ST injuries occur when the resilience of soft tissue to injury is not enough to enable athletes to tolerate the loading patterns produced during the execution of high intensity dynamic tasks (e.g., cutting, sprinting, and landing) (Kalkhoven et al., 2020). Research has shown that LE-ST injuries can have major negative consequences on a team sport athlete’s career (e.g., career termination) (Ristolainen et al., 2012) and can severely affect his/her well-being (Lohmander et al., 2007). Furthermore, when several injuries are sustained, team success (Eirale et al., 2013) and club finances can suffer (Fair and Champa, 2019; Eliakim et al., 2020). Given that the risk of sustaining a LE-ST injury can be mitigated when tailored measures are delivered, development of a validated screening model to profile injury risk would be a useful tool to help practitioners address this recurrent problem in team sports. Despite the substantive efforts made by the scientific community and sport practitioners, none of the currently available screening models (based on potential risk factors) designed to identify athletes at high risk of suffering a LE-ST injury, have adequate predictive properties (i.e., accuracy, sensitivity, and specificity) (Bahr, 2016).

Perhaps the lack of available valid screening models to predict LE-ST injuries could be attributed to the use of statistical techniques (e.g., traditional logistic regression) that have not been specifically designed to deal with class imbalance problems, such as the LE-ST injury phenomenon, in which the number of injured players (minority class) prospectively reported is always much lower than the non-injured players (majority class) (Galar et al., 2012; López et al., 2013; Fernández et al., 2017; Haixiang et al., 2017). Thus, in many scenarios including LE-ST injury, traditional screening models are often biased (for many reasons) toward the majority class (known as the “negative” class) and therefore there is a higher misclassification rate for the minority class instances (called the “positive” examples). Other issue with the current body of the literature is that the external validity of the screening models available may be limited because they are built and validated using the same date set (i.e., cohort of athletes). Apart from resulting in overly optimistic models’ performance scores, this evaluation approach does not indicate the true ability of the models to predict injuries in different data sets or cohort of athletes, which may be very low and consequently, not acceptable for injury prediction purposes. This appears to be supported by the fact that the injury predictors identified by some prospective studies have not been replicated by others using similar designs and assessment methodologies but with different samples of athletes (Croisier et al., 2002, 2008; Arnason et al., 2004; Brockett et al., 2004; Hägglund et al., 2006; Fousekis et al., 2011; Dauty et al., 2016; Timmins et al., 2016; Van Dyk et al., 2016). These limitations have led some researchers to suggest that injury prediction may be a waste of time and resources (Bahr, 2016).

In Machine Learning and Data Mining environments, some methodologies (e.g., pre-processing, cost-sensitive learning, and ensemble techniques) have been specially designed to deal with complex (i.e., non-lineal interactions among features or factors), multifactorial and class imbalanced scenarios (Galar et al., 2012; López et al., 2013; Fernández et al., 2017; Haixiang et al., 2017). These contemporary methodologies along with the use of resampling methods to assess models’ predictive power (i.e., cross-validation, bootstrap and leave-one-out) may overcome the limitations inherent to the current body of knowledge and enable the ability to build robust, interpretable, and generalizable models to predict LE-ST injuries. In fact, recent studies have used these contemporary methodologies and resampling methods as alternatives to the traditional logistic regression techniques to predict injuries in elite team sport athletes (Claudino et al., 2019). Unlike previous studies that used traditional logistic regression techniques to build prediction models (Fousekis et al., 2011; Zvijac et al., 2013; Opar et al., 2015; Hegedus et al., 2016; Van Dyk et al., 2016, 2017; Lee et al., 2018; O’Connor et al., 2020), most of these recent studies (Bartlett et al., 2017; Ge, 2017; Kautz et al., 2017; Ertelt et al., 2018; López-Valenciano et al., 2018; Rossi et al., 2018; Ayala et al., 2019), although not all (Thornton et al., 2017; Ruddy et al., 2018), have reported promising results [area under the receiver operator characteristics (AUC) scores > 0.700] to predict injuries.

However, one of the main limitations of most of these models built by the application of modern Machine Learning techniques lies in the fact that their use seems to be restricted to research settings (and not to applied environments) because sophisticated and expensive instruments (e.g., isokinetic dynamometers, force platforms, and GPS devices), qualified technicians and time-consuming testing procedures are required to collect such data. To the authors’ knowledge, there is only one study that has built a robust screening model using Machine Learning techniques (extreme gradient boosting algorithms) with data from field-based tests. Rommers et al. (2020) built a model to predict injury in elite youth soccer players based on preseason anthropometric (stature, weight, and sitting height) and motor coordination and physical fitness (strength, flexibility, speed, agility, and endurance) measures obtained through field-based tests and reported an AUC score of 0.850.

If Machine Learning techniques could build “user friendly” models with adequate predictive properties and exclusively using data obtained from questionnaires and/or cost-effective, technically undemanding, and time-efficient field-based tests, then injury prediction would not be a waste of time and resource in applied settings. In case these techniques provided a trustworthy positive response, coaches, physical trainers, and medical practitioners may know whether any of the currently available questionnaires and field-based tests to predict injuries itself works and a hierarchical rank could be developed based on their individual predictive ability of those that showed reasonably high AUC, true positive (TP), and true negative (TN) scores. Furthermore, this knowledge might be used to analyze the cost-benefit (balance between the time required to assess a single player and the predictive ability of the measures recorded) of including measures in the screening sessions for injury prediction.

Therefore, the main purpose of this study was to develop robust screening models based on pre-season measures obtained from different questionnaires and field-based tests to prospectively predict LE-ST injuries after having applied supervise Machine Learning techniques in elite male and female futsal players.

## Materials and Methods

To conduct this study, guidelines for reporting prediction model and validation studies in Health Research [Transparent Reporting of a multivariable prediction model for Individual Prognosis or Diagnosis (the TRIPOD statement)] were followed (Collins et al., 2015). The TRIPOD checklist is presented in Supplementary File 1.

### Participants

A convenience sample of 139 [72 (age: 22.5 ± 5.2 years, stature: 1.75 ± 0.7 m, body mass: 72.9 ± 6.9 kg) males and 67 (age: 22.4 ± 5.5 years, stature: 1.64 ± 0.5 m, body mass: 59.4 ± 5.1 kg) females] elite futsal players from 12 different teams [56 players (24 males and 32 females) from six club engaged in the First (top) National Spanish Futsal division and 83 players (48 males and 35 females) from six clubs engaged in the Second National Futsal division] completed this study. Elite futsal players were selected in this study because a recent published meta-analysis on injury epidemiology reported that this sport present high incidence rates of injuries (5.3 injuries per 1,000 hours of players exposure) (Ruiz-Pérez et al., 2020) and hence, urgent preventive measures are needed.

To be included in this study, all players had to be free of pain at the time of the study and currently involved in futsal-related activities. Players were excluded if: (a) they reported the presence of orthopedic problems that prevented the proper execution of one or more of the neuromuscular performance tests or (b) were transferred to another club and were not available for follow up testing at the end of 9 months. Only first injuries were used for any player sustaining multiple LE-ST injuries. The study was conducted at the end of the pre-season phase in 2015 (39 players from four teams), 2016 (44 players from four teams), 2017 (30 players from three teams), and 2018 (26 players from two teams) (September). Before any participation, experimental procedures and potential risks were fully explained to the players and coaches in verbal and written form and written informed consent was obtained from players. An Institutional Research Ethics committee approved the study protocol prior to data collection (DPS.FAR.01.14) conforming to the recommendations of the Declaration of Frontera.

### Study Design

A prospective cohort design was used to address the purpose of this study. In particular, all LE-ST injuries accounted for within the 9 months following the initial testing session (in-season phase) were prospectively collected for all players.

Players underwent a pre-season evaluation of a number of personal, psychological, self-perceived chronic ankle instability and neuromuscular performance measurements, most of them considered potential sport-related injury risk factors. In each futsal team, the testing session was conducted at the end of the pre-season phase or beginning (within the first 3 weeks) of the in-season phase of the year. The testing session was divided into three different parts. The first part of the testing session was used to obtain information related to the participants’ personal or individual characteristics. The second part was designed to assess psychological measures related to sleep quality, athlete burnout and psychological characteristics related to sport performance. The subjective perception of each player regarding his/her chronic ankle joints instability was also recorded in this second part. Finally, the third part of the session was used to assess a number of neuromuscular performance measures through three field-based tests. Each of the four testers who took part in this study had more than 6 years of experience in athletes’ screening assessment.

#### Personal or Individual Measures

The *ad hoc* questionnaire designed by Olmedilla et al. (2011) was used to record personal or individual measures that have been defined as potential non-modifiable risk factors for sport injuries: player position (goalkeeper or outfield player), current level of play (First or Second division), dominant leg (defined as the player’s kicking leg), demographic measures (sex, age, body mass, and stature) and the presence within the last season (yes or no) of LE-ST injuries with total time taken to resume full training and competition >8 days. Supplementary File 2 displays a description of the personal risk factor recorded.

#### Psychological Risk Factors

The Spanish version of the Karolinska Sleep Diary (Cervelló et al., 2014) was used to measure the sleep quality of players. The Spanish version of the Athlete Burnout Questionnaire (Arce et al., 2012) was used to assess the three different dimensions that comprise athlete burnout: (a) physical/emotional exhaustion, (b) reduced sense of accomplishment and (c) sport devaluation. The Spanish version of the Psychological Characteristics Related to Sport Performance Questionnaire designed by Gimeno et al. (2012) was used to assess five different factors: (a) stress control, (b) influence of sport evaluation, (c) motivation, (d) mental skills, and (e) group / team cohesion. Supplementary File 3 displays a description of the psychological risk factor recorded.

#### Self-Perceived Chronic Ankle Instability

The subjective perception of chronic ankle instability was measured using the Cumberland Ankle Instability Tool (CAIT). The final score was discretized into three categories of severity following the thresholds suggested by De Noronha et al. (2012): severe instability (<22 points), moderate instability (from 22 to 27 points) and minor or no instability (>27 points).

#### Neuromuscular Risk Factors

Prior to the neuromuscular risk factor assessment, all participants performed the dynamic warm-up designed by Taylor et al. (2009). The overall duration of the entire warm-up was approximately 15–20 min. The assessment of the neuromuscular risk factors was carried out 3–5 min after the dynamic warm-up.

Neuromuscular capability was determined from two different performance field-based tests: (1) isometric hip abduction and adduction strength test (Thorborg et al., 2009) and (2) Y-Balance test (dynamic postural control) (Shaffer et al., 2013). The ROM-Sport field-based battery was also carried out to assess players’ lower extremity joints range of motion (Cejudo et al., 2014).

For a matter of space, the testing maneuvers are not described below, and the reader is to refer to their original sources. Furthermore, Supplementary Files 4–6 display a description of the three field-based testing maneuvers carried and the measures recorded from each of them.

The order of the tests was consistent for all participants and was established with the intention of minimizing any possible negative influence among variables. A 5 min rest interval was given between consecutive testing maneuvers.

### Injury Surveillance

For the purpose of this study, an injury was defined as any non-contact, soft tissue (muscle, tendon, and ligament) injury sustained by a player during a training session or competition which resulted in a player being unable to take a full part in future football training or match play (Bahr et al., 2020).

These injuries were confirmed by team doctors. Players were considered injured until the club medical staff (medical doctor or physiotherapist) allowed for full participation in training and availability for match selection. Only thigh muscle (hamstrings, quadriceps, and adductors) and knee and ankle ligament injuries were considered for the analysis as these injuries are more likely to be preventable and influenced by the investigated variables.

The team medical staff of each club recorded LE-ST injuries on an injury form that was sent to the study group each month. For all LE-ST injuries that satisfied the inclusion criteria, team medical staff provided the following details to investigators: thigh muscle (hamstrings, quadriceps, and adductors), knee or ankle ligament, leg injured (dominant/non-dominant), injury severity based on lay-off time from futsal [slight/minimal (0–3 days), mild (4–7 days), moderate (8–28 days), and severe (>28 days)], date of injury, moment (training or match), whether it was a recurrence (defined as a soft tissue injury that occurred in the same extremity and during the same season as the initial injury) and total time taken to resume full training and competition. At the conclusion of the 9 month follow-up period, all data from the individual clubs were collated into a central database, and discrepancies were identified and followed up at the different clubs to be resolved. Some discrepancies among medical staff teams were found to diagnose minimal LE-ST injuries and to record their total time lost. To resolve these inconsistencies in the injury surveillance process (risk of misclassification of the players), only ST-LE injuries showing a time lost of >8 days (moderate to severe) were selected for the subsequent statistical analysis.

### Statistical Analysis

After having completed an exhaustive data cleaning process [detected anomalies or errors were removed (16 cases) and missing data (2.3%) were replaced by the mean value of the corresponding variable according to the sex (male or female) of the players] we had an imbalanced (showing an imbalance ratio of 0.22) and a high-dimensional data set comprising of 72 male and 67 female futsal players (instances) and 66 potential risk factors (features). In this study, an anomalies or error was defined as a score or value that could not be classified as real or true because of the consequence of a human error or a machine failure. An example of an error was a hip adductor PT value of 1,500 N because the measurement range of the handheld dynamometer used was from 0 to 1,335 N.

Prior to analysis, continuous data were discretized as this can improve the performance of some classifiers (Hacibeyoglu et al., 2011). Continuous variables were discretized using the unsupervised discretization algorithm available in Weka repository (Waikato Environment for Knowledge Analysis, version 3.8.3), selecting the option “optimize the number of equal-width bins” (a maximum of 10 bins were allowed per variable).

Afterward, eleven data sets were built. In particular, five data sets were built using the personal [data set (DS) 1—personal variables], psychological (DS 2—sleep quality, DS 3—athlete burnout and DS 4—psychological characteristics related to sport performance) and self-perceived (DS 5—player’s self-perceived chronic ankle joint stability) measures recorded from the questionnaires selected in this study. Likewise, three data sets were also built using the data from each of the three field-based tests carried out (DS 6—ROM-Sport battery, DS 7—isometric hip abduction and adduction strength test and DS 8—Y-Balance test). Finally, three extra data sets were built, one that grouped all the measures obtained from the questionnaires (DS 9—questionnaire-based personal, psychological, and self-perceived measures), another one that included all the neuromuscular performance measures recorded from the field-based tests (DS 10—neuromuscular performance measures from field-based tests) and finally one that contained all measures recorded (DS 11—global).

The taxonomy for learning with imbalanced data sets proposed by Galar et al. (2012) and López et al. (2013) was applied in each data set. Furthermore, this taxonomy was implemented with the approach recently proposed by Elkarami et al. (2016) because of the good results (in term of predictive performances) showed to handle imbalanced data sets (Supplementary File 7).

Four classifiers based on different paradigms, namely decision trees with C4.5 (Quinlan, 1996) and ADTree (Freund and Mason, 1999), Support Vector Machines with SMO (Gove and Faytong, 2012) and the well-known k-Nearest Neighbor (KNN) (Steinbach and Tan, 2009) as an Instance-Based Learning approach were selected. The configuration of each base classifier was optimized through the use of the metaclassifier MultiSearch.

Due to the high dimensionality of the DS 10-neuromuscular measures from field-based tests (47 variables) and DS 11-Global (66 variables), before running the algorithms included in the taxonomy just described, a feature selection process was carried out. In particular, we used the metaclassifier “attribute selected classifier” (with GreedyStepwise as search technique) available in Weka’s repository to address the feature selection process.

To evaluate the performance of the algorithms, the fivefold stratified cross-validation technique was used (Refaeilzadeh et al., 2009). The fivefold stratified cross validation was repeated a hundred times and results were averaged over the runs to obtain a more reliable estimate for the generalization ability.

The AUC and F-score were used as measures of a classifier’s performance (Altman and Bland, 1994; Zou et al., 2016). Only those algorithms whose performance scores (AUC) were higher than 0.70 were considered as acceptable for the purposes of this study and included in the intra and inter dataset comparisons analyses. Furthermore, two extra measures from the confusion matrix were also used as evaluation criteria: (a) true positive (TP) rate also called sensitivity or recall and (b) true negative (TN) rate or specificity.

In order to compare the performance of the algorithms ran in each data set (intra data set comparisons) and whose AUC scores were >0.70, the F score was selected as criterion measure. These comparisons were conducted using separate Bayesian inference analyses (Rouder et al., 2012; Lee and Wagenmakers, 2014; Wagenmakers et al., 2018). In those data sets in which (at least) a strong evidence for rejecting null hypothesis (H_0_ = no differences across algorithms’ performance scores) was found (Bayesian factor [BF_10_] > 10), a *post hoc* procedure was carried out to identify the best performing model. In the cases in which either there would not be a strong evidence for rejecting H_0_ or a group of algorithms showed the highest F-score results [without any relevant difference (BF_10_ < 10) among then], the best-performing algorithm for this dataset would be the one that showed the highest F-scores.

Finally, the best performing algorithm of each of the data sets were compared (inter dataset comparisons) using the same statistical approach in order to know which questionnaire, field-based test, or combination showed the best ability to predict moderate LE-ST injuries in elite male and female futsal players.

## Results

### Soft-Tissue Lower Extremity Injuries Epidemiology

There were 31 (16 in males and 15 in females) soft tissue injuries over the follow-up period, 17 (54.8%) of which corresponded to thigh muscles (seven hamstrings, four quadriceps, and six adductors) injuries, eight (25.8%) to knee ligament, and six (19.3%) to ankle ligament. Injury distribution between the legs was 74.1% dominant leg and 25.9% non-dominant leg. A total of 13 injures occurred during training and 18 during competition. In terms of severity, most injures were categorized as moderate (*n* = 23), whereas only eight cases were considered severe injuries (five anterior cruciate ligament injuries). Five players sustained multiple soft tissue non-contact lower extremity injuries during the observation period, so their first injury was used as the index injury in the analyses. Consequently, 25 soft-tissue injuries were finally used to develop the prediction models.

### Prediction Models for Soft Tissue Lower Extremity Injuries

All data sets are publicly available on https://data.mendeley.com/datasets/s7fs9k3nby/1. As all the algorithms selected in this study can be found in the Weka experimenter, only the scheme (and not the full code) of algorithms selected in each data set are displayed in Supplementary File 19 in order to allow practitioners to replicate our analyses and to use the models generated with their futsal players.

#### Intra-Data Set Comparisons

As displayed in the Supplementary Files 8–18, only four (DS 6—lower extremity joint ranges of motion, DS 8—dynamic postural control, DS 10—neuromuscular performance measures from field-based tests and DS 11—Global) out of 11 data sets resulted in the ability of the classification algorithms to build prediction models for LE-ST injuries with AUC scores ≥0.7.

**FIGURE 1:**
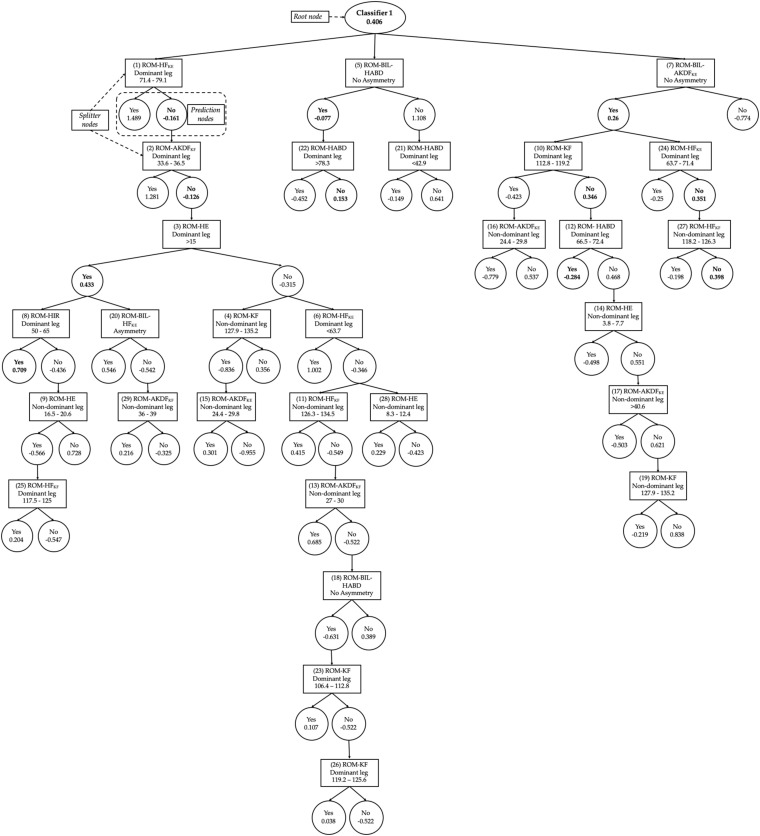
Graphical representation of the first classifier of the DS 6 (lower extremity joint ranges of motion). Prediction nodes are represented by ellipses and splitter nodes by rectangles. Each splitter node is associated with a real valued number indicating the rule condition, meaning: If the feature represented by the node satisfies the condition value, the prediction path will go through the left child node; otherwise, the path will go through the right child node. The numbers before the feature names in the prediction nodes indicate the order in which the different base rules were discovered. This ordering can to some extent indicate the relative importance of the base rules. The final classification score produced by the tree is found by summing the values from all the prediction nodes reached by the instance, with the root node being the precondition of the classifier. If the summed score is greater than zero, the instance is classified as true (low risk of LE-ST injury).

For the DS 6 - lower extremity joint ranges of motion, a total of 23 learning algorithms showed AUC scores ≥0.7. The Bayesian inference analysis carried out with these 23 algorithms (Bayesian ANOVA) reported the presence of relevant differences [BF_10_ > 100 (extreme evidence for supporting H_1_)] among their prediction performance scores. The subsequent *post hoc* analysis identified a sub-group of four algorithms whose F-scores were similar among them (F-scores ranging from 0.422 to 0.450) and also statistically higher (BF_10_ > 10) than the rest (Table 1). Among these four algorithms, the one that showed the highest F-score was the CS-Classifier technique with ADTree as base classifier (Figure 1).

**TABLE 1:** Features selected (displayed for order of importance) after having applied the classify subset evaluator filter to the data sets (DS) 10 and 11.

**Neuromuscular measures from field-based tests (DS—10)**
ROM-HF_KE_ (dominant leg)
ROM-AKDF_KE_ (dominant leg)
ROM- AKDF_KF_ (dominant leg)
ROM-BIL- HABD

**Global (DS—11)**
ROM-HF_KE_ (dominant leg)
ROM-AKDF_KE_ (dominant leg)
ROM- AKDF_KF_ (dominant leg)
ROM-BIL-HABD
Self-perceived chronic ankle instability (non-dominant leg)
History of lower extremity soft tissue injury last season

*ROM, range of motion; HF_*KE*_, *hip flexion with the knee extended; HABD, hip abduction at 90° of hip flexion; AKDF_*KE*_*, ankle dorsi-flexion with the knee extended; AKDF_*KF*_, ankle dorsi-flexion with the knee flexed; BIL, bilateral ratio.*

**FIGURE 2:**
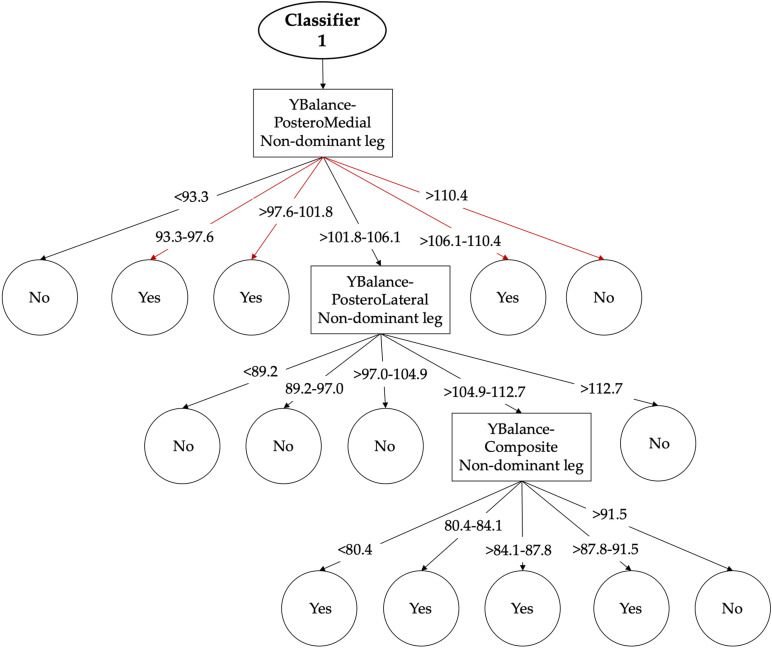
Graphical representation of the first classifier of the DS 8 (dynamic postural control). The arrows show the single pathway (transverse to the tree) through the classifier that should be followed according to participant’s scores in order to achieve a dichotomic output [high (Yes) or low (No) risk of LE-ST injury].

For its part, the DS 8—dynamic postural control only allowed to the class-balanced ensemble CS-UBAG with C4.5 as base classifier building a model with AUC scores ≥ 0.7 (AUC = 0.701 ± 0.112). In this sense, this model is comprised for 100 different C4.5 decision trees (Figure 2 shows an example of one of these C4.5 decision trees, the rest can be got upon request to the authors).

**FIGURE 3:**
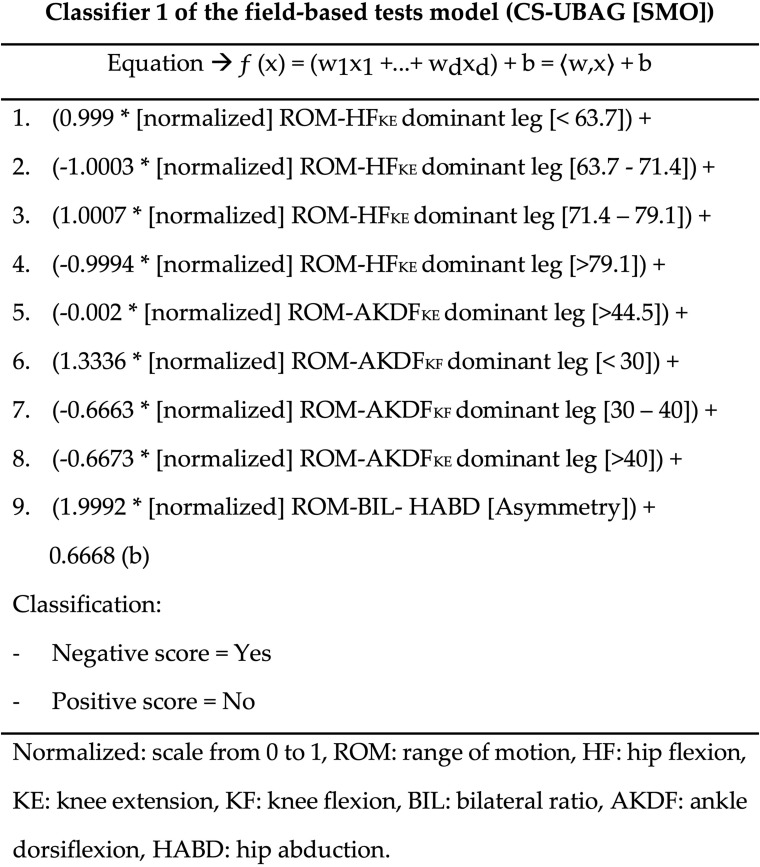
Description of the first classifier of the DS 10 (field-based tests).

The feature selection process carried out in the DS 10—neuromuscular measures from field-based tests identified a subset of four ROM measures as the most relevant (considering the individual predictive ability of each feature along with the degree of redundancy among them) on which was subsequently applied the taxonomy of learning algorithms described in the “Materials and Methods” section. Thus, a total of 66 algorithms built (using this subset of features) prediction models with AUC scores ≥0.7. The Bayesian analysis conducted with these 66 algorithms documented the existence of relevant differences [with an extreme degree of evidence (BF_10_ > 100)] among their predictive ability scores. The subsequent *post hoc* analysis reported that a group of three algorithms showed similar F-scores among them (ranging from 0.458 to 0.474) but significantly higher than the rest. Therefore, the selection of the best performing algorithm of this DS 10 was based on the highest F-score. Thus, the algorithm CS-UBAG with SMO as base classifier was the one that showed the highest F-score (0.474 ± 0.111) and hence, it was selected for the inter data set comparisons. Figure 3 displays an example of the 100 predictors than this prediction model is comprised (the rest can be got upon request to the authors).

**FIGURE 4:**
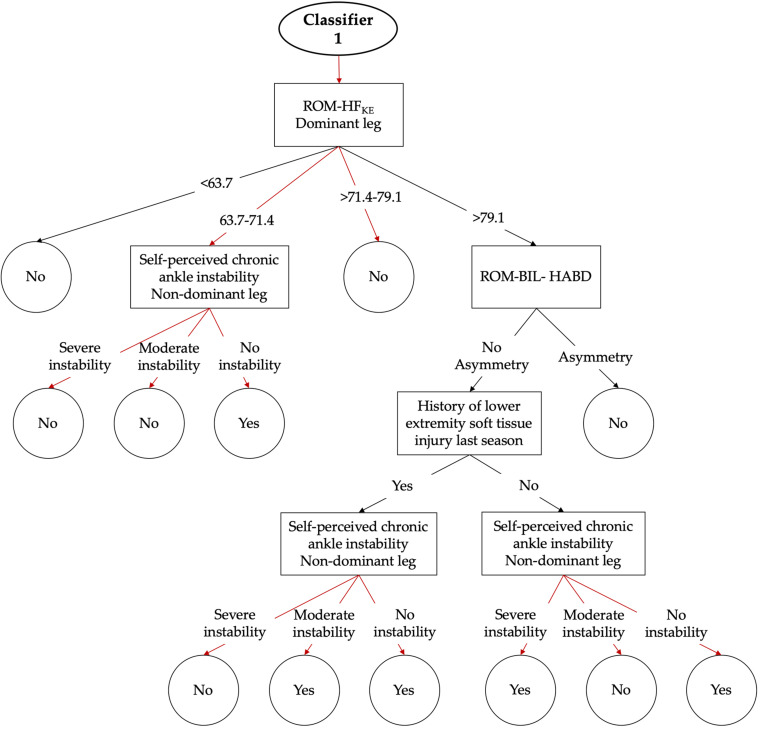
Graphical representation of the first classifier of the DS 11 (global). The arrows show the single pathway (transverse to the tree) through the classifier that should be followed according to participant’s scores in order to achieve a dichotomic output [high (Yes) or low (No) risk of LE-ST injury].

The DS 11, that comprised of the 66 personal (*n* = 8), psychological (*n* = 9), self-perceived chronic ankle instability (*n* = 2) and neuromuscular performance (47) features was reduced to a subset of six features by the feature selection metaclassifier selected, from which four were ROM measures, one was a self-perceived chronic ankle instability measure and the last one belonged to the group of personal measures (Table 2). This sub-set of features allowed 59 algorithms building prediction models showing AUC scores ≥0.7. Finally, and it is showed in the Table 1, the Bayesian inference and the subsequent *post hoc* analyses identified the class-balanced ensemble CS-UBAG with C4.5 as base classifier as the best-performing algorithm (AUC = 0.749 ± 0.105, TP rate = 75.5% ± 23.6, TN rate = 62.7 ± 11.5, F-score = 0.436 ± 0.122). An example of the 100 C4.5 decision trees that comprised this model is presented in Figure 4.

**TABLE 2:** Best-performing sub-set of algorithms for those data sets (DS) that allowed building prediction models with AUC scores ≥0.7.

**Technique**	**Performance measures**			
	**AUC**	**TP rate (%)**	**TN rate (%)**	**F-score**

		**Lower extremity joint ranges of motion (DS—6)**		
ADTree	0.754 ± 0.122	35.8 ± 21.6	93.4 ± 6.3	0.433 ± 0.195
ROS (ADTree)	0.745 ± 0.126	46.1 ± 23.5	87.4 ± 8.3	0.442 ± 0.188
**CS-Classifier (ADTree)**	**0.757 ± 0.124**	**44.7 ± 23.2**	**89.1 ± 8.4**	**0.450 ± 0.184**
CS-UBAG (ADTree)	0.737 ± 0.106	48.3 ± 21.5	83.0 ± 8.1	0.422 ± 0.161

	**Dynamic postural control (DS—8)**			

**CS-UBAG (C4.5)**	**0.701 ± 0.114**	**64.9 ± 21.1**	**63.3 ± 10.4**	**0.388 ± 0.109**

	**Neuromuscular measures from field-based tests (DS—10)**			

CS-OBAG (SMO)	0.760 ± 0.103	83.3 ± 22.9	62.9 ± 10.0	0.469 ± 0.115
CS-UBAG (C4.5)	0.748 ± 0.089	87.6 ± 20.3	57.2 ± 10.7	0.458 ± 0.100
**CS-UBAG (SMO)**	**0.767 ± 0.096**	**85.1 ± 21.4**	**62.1 ± 9.8**	**0.474 ± 0.111**

	**Global (DS—11)**			

OBAG (SMO)	0.742 ± 0.125	51.3 ± 25.5	79.5 ± 9.6	0.410 ± 0.179
UBAG (SMO)	0.737 ± 0.121	54.7 ± 25.6	76.3 ± 10.2	0.410 ± 0.171
CS-OBAG (C4.5)	0.751 ± 0.107	60.9 ± 28.2	73.2 ± 10.6	0.418 ± 0.163
CS-OBAG (SMO)	0.747 ± 0.121	65.1 ± 27.9	70.1 ± 11.3	0.423 ± 0.151
**CS-UBAG (C4.5)**	**0.749 ± 0.105**	**75.5 ± 23.6**	**62.7 ± 11.5**	**0.436 ± 0.122**
CS-UBAG (ADTree)	0.741 ± 0.119	62.0 ± 27.3	72.0 ± 10.4	0.419 ± 0.161
CS-UBAG (SMO)	0.747 ± 0.116	70.8 ± 26.1	66.5 ± 10.9	0.433 ± 0.137
CS-UBAG (IBK)	0.722 ± 0.124	71.8 ± 23.9	61.6 ± 12.3	0.413 ± 0.122
CS-SBAG (C4.5)	0.755 ± 0.115	55.7 ± 28.2	76.2 ± 11.0	0.409 ± 0.175
CS-SBAG (SMO)	0.750 ± 0.121	58.4 ± 27.2	74.7 ± 11.1	0.416 ± 0.164

*Highlighted in bold are the algorithms selected in each DS for the posterior inter-group comparative analysis.*

*AUC, area under the ROC curve; TP rate, true positive rate; TN rate, true negative rate.*

#### Inter-Data Set Comparisons

The inter data set comparison analysis carried out with the best-performing algorithms of the DS 6 [CS-Classifier (ADTree)], 8 [CS-UBAG (C4.5)], 10 [CS-UBAG (SMO)] and 11 [CS-UBAG (C4.5)] showed that the algorithm of the DS 8 obtained significantly lower F-scores than the other three algorithms (BF_10_ > 100). However, there were no statistically differences among the algorithms from the DS 6, 10, and 11. Among these three algorithms, the one from the DS 10 demonstrated the highest F-score and was considered as the “winning model” (Table 2). Models from DS 8, 10, and 11 are comprised by 100 classifiers. In term of practical applications, each classifier has a vote or decision [yes (high risk of LE-ST injury) or no (lower risk of LE-ST injury)], and the final decision regarding whether or not a player might suffer an injury is based on the combination of the votes of each individual classifier to each class (yes or no).

## Discussion

The main findings of this study indicate that only those groups of measures from two of the field-based tests [ROM-Sport battery (AUC = 0.751 ± 0.124) and Y-Balance (AUC = 0.701 ± 0.114)], as independent data sets, can build robust models (AUC ≥ 0.7) to identify elite futsal players at risk of sustaining a LE-ST injury. One of the possible reasons why only the lower extremity ROM and dynamic postural control measures can separately build robust prediction models may be related to the fact that they play a significant role in the hazardous lower extremity movement patterns performed by futsal players. In particular the execution of numerous weight-bearing high intensity locomotive actions (e.g., cutting, landing, and sprinting) that may produce excessive dynamic valgus at the knee with limited hip and knee flexion ROMs, which have been identified as primary and modifiable LE-ST injury patterns (Robinson and Gribble, 2008; Thorpe et al., 2008; Lockie et al., 2013; Ambegaonkar et al., 2014; Booysen et al., 2015; Overmoyer and Reiser, 2015). The fact that the best-performing model built with the ROM data set (DS 6) showed a significantly higher prediction performance [and also less decision trees (1 vs. 100)] than its counterpart model built with the dynamic postural control data set (DS 7) (F-score = 0.450 vs. 0.388) may be due to the fact that the scores obtained thorough the Y-Balance test are widely influenced by hip and knee flexion and the ankle dorsiflexion ROM measures in the sagittal plane and to less extend by dynamic core stability (in the frontal plane) and isokinetic knee flexion strength measures (Ruiz-Pérez et al., 2019). Thus, the dynamic postural control measures obtained from the Y-Balance test might have allowed the construction of a model with an acceptable prediction ability mainly due to the influence of whole lower limb posterior kinetic chain ROMs in the distances reached. This hypothesis may also be supported by the fact that the feature selection process carried out in the data set in which all the neuromuscular performance measures were grouped (DS 10) and also in the data set that contained all the measures recorded in this study (DS 11) did not consider any of the dynamic postural control measures in contrast to the hip flexion and ankle dorsiflexion ROM measures that were considered LE-ST injury predictors.

Previous studies have explored the individual predictive ability of some (but not many) field-based tests [e.g., Y-Balance (Butler et al., 2013), leg squat (O’Connor et al., 2020), side plank (Hegedus et al., 2016), and drop jump (Myer et al., 2010, 2011)] to identify athletes from intermittent team sports at high risk of LE-ST injury using traditional logistic regression techniques. Most of these studies have reported models exhibiting high sensitivity values (TN rates) but very low specificity values (TP rates) and hence, cannot be used for injury prediction. For example, O’Connor et al. (2020) examined whether a standardized visual assessment of squatting technique and core stability can predict lower extremity injuries in a large sample of collegiate Gaelic players (*n* = 627). The logistic regression-based model generated revealed that while the TP rate was moderate to high (76%) the TN rate was low (44%). This circumstance reflects one of the main limitations inherent in traditional regression techniques, that is to say, they do not deal well with imbalanced data sets [their models usually are biased toward the majority class (true negative rates) to optimize the percentage of well-classified instances] (Galar et al., 2012). Furthermore, the validation technique applied to the models generated in these studies may not be exigent enough to ensure that the phenomenon of over-fitting was minimized as the models were validated using the data from the population with whom the prediction equations were generated (Bahr, 2016; Jovanovic, 2017).

Due to their high cost (approximately 250€ per unit) currently available GPS systems may not be considered as accessible tools for most practitioners that work in applied sport settings, however, it should be noted that prediction models to identify team sport athletes (mainly soccer and rugby players) at risk of sustaining a LE-ST injury based exclusively on external training workload measures and built using learning algorithms are available (Bartlett et al., 2017; Thornton et al., 2017; Rossi et al., 2018). However, only the model reported by Rossi et al. (2018) has shown AUC scores ≥0.7 after 16 weeks of data collection (AUC = 0.760). The predictive ability of the model built by Rossi et al. (2018) is very similar to the predictive ability shown in our best-performing prediction model built using only lower extremity ROM measures (AUC = 0.757). Nevertheless, our prediction model based on ROM measures has a higher external validity for practitioners in applied environments due to two main aspects. Firstly, the low cost of the materials needed to conduct the assessment maneuvers (inclinometer with a telescopic arm = 200€, lumbar protection support = 50€). Secondly, our model was developed and validated using ROM measures from 139 elite futsal players from 12 different teams, whereas Rossi et al. (2018) only assessed the external training workload of 26 elite soccer players all from the same team. Consequently, the model displayed by Rossi et al. (2018) can only be used by the medical and performance staff of the team in which the external workload measures were collected due (among other factors) to the high inter-team differences in training and competitive calendars, drills prescribed in training sessions and tactical systems adopted throughout match play.

The results of this study also reported that the combination in the same data set (DS 9) of all the measures obtained from the five questionnaires selected did not permit classification algorithms to build prediction models with acceptable performance scores (AUC scores ranged from 0.443 to 0.558). Previous studies have documented the existence of significant associations between some personal characteristics [e.g., age (Arnason et al., 2004; Hägglund et al., 2006; Dauty et al., 2016) and recent history of injury (Brockett et al., 2004; Hägglund et al., 2006; López-Valenciano et al., 2018; Ayala et al., 2019)], psychological constructs [e.g., physical/emotional exhaustion, reduce sense of accomplishment, sports devaluation (Cresswell and Eklund, 2006; Moen et al., 2016)] and self-perceived chronic ankle instability (Hiller et al., 2006, 2011), sleep quality (López-Valenciano et al., 2018; Palucci Vieira et al., 2020) measures, and LE-ST injury. However, it may be possible that the magnitude of these associations between the questionnaire-based measures and LE-ST injury, neither individually nor collectively, are strong enough to build robust models with the aim of identifying elite futsal players at risk of LE-ST injury. On the contrary, the grouping in the same data set (DS 10) of all the neuromuscular performance measures obtained from the three field-based tests did permit prediction models to be built with moderate performance scores (AUC ≥ 0.7). The feature selection technique applied to this data set with the aim of reducing its dimensionality (46 features) through deleting redundant and not relevant measures (considered as noise) only selected four ROM measures, with whom the CS-UBAG method with SMO as base classifier built a prediction model with AUC and F-scores of 0.767 and 0.474, respectively. This model reported the highest performance scores, together with the fact that only two hip and two ankle ROM measures are needed to run the screen in a single player making it appropriate for applied scenarios. Finally, the inclusion in the same data set (DS 11) of all the eight groups of measures obtained from the five questionnaires and three field-based tests did not result in models with significantly higher performance scores and hence, the null hypothesis was rejected.

The prediction properties of the “model of best fit” of the current study were lower than that reported by the only other study that has used Machine Learning techniques to develop a screening model based on field-based measures (AUC = 0.767 vs. 0.850, TP rate = 85 vs. 85%, TN rate = 62 vs. 85%) (Rommers et al., 2020). One of the potential reasons that may explain this difference in models’ predictive performance in favor of Rommers et al.’s 2020 model can be attribute to its higher sample size (734 elite young soccer players vs. 139 elite adult futsal players) and the less rigorous resampling technique applied in its validation process [hold out with 20% of the sample (test data set) vs. fivefolds stratified cross validation]. Although the predictive properties of our model are lower than Rommers et al.’s 2020 model (but they are acceptable for an injury prediction standpoint), it should be highlighted that only four ROM measures and 5 min are needed to run the screen in a single player, unlike Rommers et al.’s 2020 model that requires 20 measures obtained from a questionnaire and five different field-based tests, which can take longer than 45 min to collect all of them in a single player.

The current study has a number of limitations that must be acknowledged. The first potential limitation of the current study is the population used. The sport background of participants was elite futsal and the generalizability to other sport modalities and level of play cannot be ascertained. Although all the measures recorded during the screening session are purported as LE-ST injury risk factors, there are a number of other measures from different questionnaires and field-based tests not included in this study (due to time constraints) which have been associated with LE-ST injury (e.g., back extensor and flexor endurance measures, bilateral leg strength asymmetries, relative leg stiffness and reactive strength index) and that may have improved the ability to predict LE-ST injuries in this cohort of athletes. Neither situational (e.g., pressing and tackling, regaining balance after kicking, side-stepping, and landing from a jump) nor movement (e.g., excessive dynamic knee valgus motion at the knee, limited hip, and knee flexion angles) patterns for those futsal players who suffered a LE-ST injury were recorded for this study due to technical reasons (i.e., training sessions and matches were not recorded and hence, a systematic biomechanical/kinematic video analysis on injury patterns was not possible to be conducted). Although the main findings of this study may help identify futsal players at high risk of LE-ST injury, having included information regarding situational and movement injury patterns in the models might have not only increase their predictive performance scores but shed light on why and how LE-ST injuries occur in futsal players. Despite the fact that the number of both futsal players assessed (*n* = 139) and LE-ST injuries recorded (*n* = 25) was large enough to build robust prediction models, the inclusion of more instances in the learning processes of the models may have improved their performance scores. Finally, out of the 8^8^ possible combinations of measures that could have been analyzed with the data from the five questionnaires and three field-based tests, only three of them were explored, from both a time perspective and based on those that would be most interesting from a practitioner perspective. Therefore, it is unknown if other combinations of measures, different from the ones analyzed in this study, may have provided prediction models with higher AUC scores.

## Conclusion

In conclusion, thanks to the application of novel machine learning techniques, the current study has developed four screening models based on field-based measures (mainly ROM and dynamic postural control features) that showed moderate accuracy (AUC scores ranged from 0.701 to 0.767, determined all through the exigent cross-validation resampling technique) for identifying elite futsal players at risk of LE-ST injury. The “model of best fit” of the current study (AUC = 0.767, TP rate = 85% and TN rate = 62%) was comprised by only two hip (flexion with knee extended and abduction) and two ankle (dorsiflexion with knee flexed and extended) ROM measures and 10 different classifiers. Given that these ROM measures require little equipment to be recorded and can be employed quickly (approximately 5 min) and easily by trained staff in a single player, the model developed in this study should be included as an essential component of the injury management strategy in elite futsal.

## Data Availability Statement

Data is available online at: https://data.mendeley.com/datasets/s7fs9k3nby/2.

## Ethics Statement

The studies involving human participants were reviewed and approved by Órgano evaluador de proyectos, Universidad Miguel Hernández de Elche (DPS.FAR.02.14). The patients/participants provided their written informed consent to participate in this study.

## Author Contributions

All authors listed have made a substantial, direct and intellectual contribution to the work, and approved it for publication.

## Conflict of Interest

The authors declare that the research was conducted in the absence of any commercial or financial relationships that could be construed as a potential conflict of interest.

## Supplementary Material

Supplementary File 1 TRIPOD checklist: prediction model development and validation.

Supplementary File 2 Description of the personal or individual injury risk factors recorded.

Supplementary File 3 Description of the psychological risk factors recorded.

Supplementary File 4 Description of the testing maneuver and measures obtained from the isometric hip abduction and adduction strength test.

Supplementary File 5 Description of the testing maneuver and measures obtained from the Y-Balance test.

Supplementary File 6 Description of the testing maneuver and measures obtained from the ROM-Sport battery.

Supplementary File 7 Descriptions of the resampling, ensemble and cost-sensitive algorithms applied to the base classifiers.

Supplementary File 8 AUC results (mean and standard deviation) of the personal or individual characteristics data set (DS 1) for the five base classifiers in isolation and after applying in them the resampling, ensemble (Classic, Boosting-based, Bagging-based, and Class-balanced ensembles) and cost-sensitive learning techniques selected.

Supplementary File 9 AUC results (mean and standard deviation) of the sleep quality data set (DS 2) for the four base classifiers in isolation and after applying in them the resampling. ensemble (Classic, Boosting-based, Bagging-based, and Class-balanced ensembles) and cost-sensitive learning techniques selected.

Supplementary File 10 AUC results (mean and standard deviation) of the Athlete Burnout data set (DS 3) for the four base classifiers in isolation and after applying in them the resampling ensemble (Classic, Boosting-based, Bagging-based, and Class-balanced ensembles) and cost-sensitive learning techniques selected.

Supplementary File 11 AUC results (mean and standard deviation) of the psychological characteristics related to sport performance data set (DS 4) for the four base classifiers in isolation and after applying in them the resampling ensemble (Classic, Boosting-based, Bagging-based, and Class-balanced ensembles) and cost-sensitive learning techniques selected.

Supplementary File 12 AUC results (mean and standard deviation) of the self-perceived chronic ankle instability data set (DS 5) for the four base classifiers in isolation and after applying in them the resampling. ensemble (Classic, Boosting-based, Bagging-based, and Class-balanced ensembles) and cost-sensitive learning techniques selected.

Supplementary File 13 AUC results (mean and standard deviation) of the lower extremity joint ranges of motion data set (DS 6) for the five base classifiers in isolation and after applying in them the resampling, ensemble (Classic, Boosting-based, Bagging-based, and Class-balanced ensembles) and cost-sensitive learning techniques selected.

Supplementary File 14 AUC results (mean and standard deviation) of the isometric hip abduction and adduction strength data set (DS 7) for the five base classifiers in isolation and after applying in them the resampling, ensemble (Classic, Boosting-based, Bagging-based, and Class-balanced ensembles) and cost-sensitive learning techniques selected.

Supplementary File 15 AUC results (mean and standard deviation) of the dynamic postural control data set (DS 6) for the five base classifiers in isolation and after applying in them the resampling, ensemble (Classic, Boosting-based, Bagging-based, and Class-balanced ensembles), and cost-sensitive learning techniques selected.

Supplementary File 16 AUC results (mean and standard deviation) of the measures obtained through questionnaires data set (DS 6) for the five base classifiers in isolation and after applying in them the resampling, ensemble (Classic, Boosting-based, Bagging-based, and Class-balanced ensembles), and cost-sensitive learning techniques selected.

Supplementary File 17 AUC results (mean and standard deviation) of the field-based tests of neuromuscular performance data set (DS 6) for the five base classifiers in isolation and after applying in them the resampling, ensemble (Classic, Boosting-based, Bagging-based, and Class-balanced ensembles) and cost-sensitive learning techniques selected.

Supplementary File 18 AUC results (mean and standard deviation) of the global data set (DS 11) for the five base classifiers in isolation and after applying in them the resampling, ensemble (Classic, Boosting-based, Bagging-based, and Class-balanced ensembles) and cost-sensitive learning techniques selected.

Supplementary File 19 Schemes of the algorithms selected in data sets (DS) 6, 8, 10, and 11.
