# Supplementary material for: A Field-Based Approach to Determine Soft Tissue Injury Risk in Elite Futsal Using Novel Machine Learning Techniques
Source: Front Psychol. 2021 Feb 5;12:610210. doi: 10.3389/fpsyg.2021.610210 (PMC7892460; doi:10.3389/fpsyg.2021.610210)
Supplement: Supplementary File 2 — Description of the personal or individual injury risk factors recorded. [file Table_2.DOCX]

| **Supplementary file 2.** Description of the personal or individual injury risk factors recorded | |
| --- | --- |
| **Name** | **Labels** |
| Player position | Goalkeeper or outfield player |
| Current level of play | 1^st^ division or 2^nd^ division |
| Dominant leg | Right, left or two-footed |
| Sex | Male or female |
| Age | Sub21, sub23, senior (23-30 y) or veteran (> 30y) |
| Body mass (kg) | <50, 50-54.1, >54.1-58.2, >58.2-62.3, >62.3-66.4, >66.4-70.5 or >70.5 |
| Stature (cm) | <148.5, 148.5-156.1, >156.1-163.7, >163.7-171.2, >171.2-178.8, >178.8-186.4 or >186.4 |
| History of lower extremity soft tissue injury last season | Yes or no |
