# Supplementary material for: A Field-Based Approach to Determine Soft Tissue Injury Risk in Elite Futsal Using Novel Machine Learning Techniques
Source: Front Psychol. 2021 Feb 5;12:610210. doi: 10.3389/fpsyg.2021.610210 (PMC7892460; doi:10.3389/fpsyg.2021.610210)
Supplement: Supplementary File 3 — Description of the psychological risk factors recorded. [file Table_3.DOCX]

| **Supplementary file 3.** Description of the psychological risk factors recorded | |
| --- | --- |
| **Name** | **Labels** |
| Sleep quality | <2.46, 2.46-3.02, >3.02-3.58, >3.58-4.14 or >4.14 |
| **Athlete Burnout** | |
| a) Physical/emotional exhaustion | <1.5, 1.5-1.8, >1.8-2.1, >2.1-2.4 or >2.4 |
| b) Reduced sense of accomplishment | <2.1 or >2.1 |
| c) Sport devaluation | <1.3, 1.3-1.6, >(1.6-1.9, >1.9-2.2, >2.2-2.5, >2.5-2.8, >2.8-3.1, >3.1-3.4, >3.4-3.7 or >3.7 |
| **Psychological Characteristics Related to Sport Performance** | |
| 1. Stress control | <30.8, 30.8-42.6, >42.6-54.4, >54.4-66.2 or >66.2 |
| 1. Influence of sport evaluation | <20.8, >20.8-23.6, >23.6-26.4, >26.4-29.2, >29.2-32, >32-34.8 or >34.8 |
| 1. Mental skills | <13, 13-15, >15-17, >17-19, >19-21, >21-23 or >23 |
| 1. Motivation | <13.1, 13.1-15.2, >15.2-17.3, >17.3-19.4, >19.4-21.5, >21.5-23.6, >23.6-25.7 or >25.7 |
| 1. Team cohesion | <17, 17-23 or >23 |
