# Supplementary material for: A Field-Based Approach to Determine Soft Tissue Injury Risk in Elite Futsal Using Novel Machine Learning Techniques
Source: Front Psychol. 2021 Feb 5;12:610210. doi: 10.3389/fpsyg.2021.610210 (PMC7892460; doi:10.3389/fpsyg.2021.610210)
Supplement: Supplementary File 4 — Description of the testing maneuver and measures obtained from the isometric hip abduction and adduction strength test. [file Table_4.DOCX]

| **Supplementary file 4.** Description of the measures obtained from the isometric hip abduction and adduction strength test | | |  |
| --- | --- | --- | --- |
| **Name** | **Labels** | |  |
|  | **Dominant Leg** | **Non-Dominant Leg** | |
| PT_ISOM_-HipAbd-Normalized | <1.64, 1.64-1.89, >1.89-2.14, >2.14-2.39, >2.39-2.63, >2.63-2.88 or >2.88 | <1.85, 1.85-2.17, >2.17-2.5, >2.5-2.83, >2.83-3.16, >3.16-3.48 or >3.48 | |
| PT_ISOM_-HipAdd- Normalized | <1.57, 1.57-1.84, >1.84-2.11, >2.11-2.37, >2.37-2.63, >2.63-2.9 or >2.9 | <1.58, 1.58-1.86, >1.86-2.14, >2.14-2.42 or >2.42 | |
| UnRatio-ISOM-HipAbd/HipAdd | <0.74, 0.74-0.82, >0.82-0.91, >0.91-0.99, >0.99-1.08, >1.08-1.17, >1.17-1.25, >1.25-1.34, >1.34-1.42 or >1.42 | <0.69, 0.69-0.83, >0.83-0.97, >0.97-1.11, >1.11-1.24 or >1.24 | |
| BilaRatio-PT_ISOM_-HipAbd | No Asymmetry or Asymmetry | |  |
| BilaRatio-PT_ISOM_-HipAdd | No Asymmetry or Asymmetry | |  |
| Bila: bilateral; Uni: unilateral; ISOM: isometric; PT: peak torque; Abd: abduction; Add: adduction. | | |  |
