# Supplementary material for: A Field-Based Approach to Determine Soft Tissue Injury Risk in Elite Futsal Using Novel Machine Learning Techniques
Source: Front Psychol. 2021 Feb 5;12:610210. doi: 10.3389/fpsyg.2021.610210 (PMC7892460; doi:10.3389/fpsyg.2021.610210)
Supplement: Supplementary File 5 — Description of the testing maneuver and measures obtained from the Y-Balance test. [file Table_5.DOCX]

| **Supplementary file 5.** Description of the measures obtained from the Y-Balance test | | | |
| --- | --- | --- | --- |
| **Name** | **Labels** | | |
|  | **Dominant Leg** | | **No Dominant Leg** |
| Y-Balance-Anterior | <50.9, 50.9-55.7, >55.7-60.5, >60.55-65.4, >65.4-70.2 or >70.2 | | <51.3, 51.3-56.7, >56.7-62.2, >62.2-67.7, >67.7-73.1 or >73.1 |
| Y-Balance-PosteroMedial | <83.1, 83.1-88.7, >88.7-94.4, >94.4-100.1, >100.1-105.8, >105.8-111.4 or >111.4 | | <93.3, 93.3-97.6, >97.6-101.8, >101.8-106.1, >106.1-110.4 or >110.4 |
| Y-Balance-PosteroLateral | <81.7, 81.7-91.4, >91.4-101.1, >101.1-110.7 or >110.7 | | <89.2, 89.2-97.0, >97.0-104.9, >104.9-112.7 or >112.7 |
| BilaRatio-Y-Balance-Anterior | No Asymmetry or Asymmetry | | |
| BilaRatio-Y-Balance-PosteroMedial | No Asymmetry or Asymmetry | | |
| BilaRatio-Y-Balance-PosteroLateral | No Asymmetry or Asymmetry | | |
| Y-Balance-Composite | <78.4, 78.4-85.9, >85.9-93.3 or >93.3 | <80.4, 80.4-84.1, >84.1-87.8, >87.8-91.5 or >91.5 | |
