# Supplementary material for: A Field-Based Approach to Determine Soft Tissue Injury Risk in Elite Futsal Using Novel Machine Learning Techniques
Source: Front Psychol. 2021 Feb 5;12:610210. doi: 10.3389/fpsyg.2021.610210 (PMC7892460; doi:10.3389/fpsyg.2021.610210)
Supplement: Supplementary File 6 — Description of the testing maneuver and measures obtained from the ROM-Sport battery. [file Table_6.DOCX]

| **Supplementary file 6.** Description of the measures obtained from the lower extremity range of motion assessment tests | | |
| --- | --- | --- |
| **Name** | **Labels** | |
|  | **Dominant Leg** | **Non-Dominant Leg** |
| ROM-HF_KF_ | <117.5, 117.5-125, >125-132.5, >132.5-140, >140-147.5 or >147.5 | <118.2, 118.2-126.3, >126.3-134.5, >134.5-142.7, >142.7-150.8 or >150.8 |
| ROM-HF_KE_ | <63.7, 63.7-71.4, >71.4-79.1 or >79.1 | <59, 59-68 or >68 |
| ROM-HE | <0.1, 0.1-3.8, >3.8-7.7, >7.7-11.6, >11.6-15.5 or >15.5 | <0.1, 0.1-4.2, >4.2-8.3, >8.3-12.4, >12.4-16.5, >16.5-20.6 or >20.6 |
| ROM-HABD | <42.9, 42.9-48.8, >48.8-54.7, >54.7-60.6, >60.6-66.5, >66.5-72.4, >72.4-78.3 or >78.3 | <46.5, 46.5-67, >67-87.5 or >87.5 |
| ROM-HIR | <35, 35-50, >50-65 or >65 | <30.9, 30.9-36.8, >36.8-42.7 or >42.7 |
| ROM-HER | <40.8, 40.8-50.6, >50.6-60.4, >60.4-70.2 or >70.2 | <42.8, 42.8-54.6, >54.6-66.4, >66.4-78.2 or >78.2 |
| ROM-KF | <106.4, 106.4-112.8, >112.8-119.2, >119.2-125.6, >125.6-132, >132-138.4, >138.4-144.8 or >144.8 | <98.4, 98.4-105.7, >105.7-113.1, >113.1-120.5, >120.5-127.9, >127.9-135.2, >135.2-142.6 or >142.6 |
| ROM-AKDF_KE_ | <44.5 or >44.5 | <24.4, 24.4-29.8, >29.8-35.2, >35.2-40.6 or >40.6 |
| ROM- AKDF_KF_ | <24.9, 24.9-27.8, >27.8-30.7, >30.7-33.6, >33.6-36.5, >36.5-39.4, >39.4-42.3 or >42.3 | <24, 24-27, >27-30, >30-33, >33-36, >36-39 or >39 |
| ROM-BIL- HF_KF_ | No Asymmetry or Asymmetry | |
| ROM-BIL- HF_KE_ | No Asymmetry or Asymmetry | |
| ROM-BIL- HE | No Asymmetry or Asymmetry | |
| ROM-BIL- HABD | No Asymmetry or Asymmetry | |
| ROM-BIL- HIR | No Asymmetry or Asymmetry | |
| ROM-BIL- HER | No Asymmetry or Asymmetry | |
| ROM-BIL- KF | No Asymmetry or Asymmetry | |
| ROM-BIL- AKDF_KE_ | No Asymmetry or Asymmetry | |
| ROM-BIL- AKDF_KF_ | No Asymmetry or Asymmetry | |
| ROM: range of motion; HF_KF_: hip flexion with the knee flexed; HF_KE_: hip flexion with the knee extended; HE: Hip extension; HABD: hip abduction at 90º of hip flexion; HIR: hip internal rotation; HER: hip external rotation; KF: knee flexion; AKDF_KE_: ankle dorsi-flexion with the knee extended; AKDF_KF_: ankle dorsi-flexion with the knee flexed; BIL: bilateral ratio. | | |
