# Supplementary material for: A Field-Based Approach to Determine Soft Tissue Injury Risk in Elite Futsal Using Novel Machine Learning Techniques
Source: Front Psychol. 2021 Feb 5;12:610210. doi: 10.3389/fpsyg.2021.610210 (PMC7892460; doi:10.3389/fpsyg.2021.610210)
Supplement: Supplementary File 7 — Descriptions of the resampling, ensemble and cost-sensitive algorithms applied to the base classifiers. [file Table_7.DOCX]

**Supplementary file 7.** Dascriptions’ of the resampling, ensemble and cost-sensitive algorithms applied to the base classifiers.

With regard to the resampling techniques, four (two oversampling and two undersampling algorithms) of the most popular methodologies were selected, which are the synthetic minority oversampling technique (SMOTE)^257^, random oversampling (ROS), random undersampling (RUS) and Wilson’s edited nearest neighbor rule (ENN)^258^. In the four resampling techniques selected, a level of balance in the training data near the 40/60 was attempted. In addition, the interpolations that are computed to generate new synthetic data are made considering the k-5-nearest neighbors of minority class instances using the Euclidean distance.

Regarding ensemble learning algorithms, classic ensembles such as Bagging^259^, AdaBoost^260^ and AdaBoot.M1^261^ were included in this study. Furthermore, the algorithm families designed to deal with skewed class distributions in data sets were also included: Boosting-based and Bagging-based. The Boosting based ensembles that were considered in the current study were SMOTEBoost^262^ and RUSBoost^263^. Concerning Bagging based ensembles, it was included from the OverBagging group, OverBagging (which uses ROS)^264^, UnderBagging (which uses RUS)^264^ and SMOTEBagging^264^. The number of internal classifiers used within each ensemble learning algorithm was set 100 (always the same) base classifiers (C4.5, ADTree, SVM and KNN) by default.

Concerning the cost-sensitive learning algorithms, two different algorithms were used, namely MetaCost^265^ and cost-sensitive classifier. Cost-sensitive learning solutions incorporating both the data (external) and algorithmic level (internal) approaches assume higher misclassification costs for samples in the minority class and seek to minimize the high cost errors. For the both cost-sensitive algorithms selected, the cox matrix set-up was to:

c = $\left\{ \begin{matrix} 0 & 2 \\ 1 & 0 \end{matrix} \right\}$ where a false negative has a cost of 2 and false positive had a cost of 1.

The behavior of some specific combinations of class-balanced ensembles with cost-sensitive base classifiers was also studied. Finally, the algorithm Random Forest^266^ in isolation and in combination with the resampling techniques was also explored due to its good results showed in previous studies^267^.

For the sake of brevity and the lack of space, the code of the algorithms used in this study has not been written here. Instead, we have only specified the names and refer the reader to their original sources. Furthermore, all the classification algorithms used are available in Weka Data Mining software.
