# Supplementary material for: A Field-Based Approach to Determine Soft Tissue Injury Risk in Elite Futsal Using Novel Machine Learning Techniques
Source: Front Psychol. 2021 Feb 5;12:610210. doi: 10.3389/fpsyg.2021.610210 (PMC7892460; doi:10.3389/fpsyg.2021.610210)
Supplement: Supplementary File 8 — AUC results (mean and standard deviation) of the personal or individual characteristics data set (DS 1) for the five base classifiers in isolation and after applying in them the resampling, ensemble (Classic, Boosting-based, Bagging-based, and Class-balanced ensembles) and cost-sensitive learning techniques selected. [file Table_8.DOCX]

| **Supplementary file 8.** AUC results (mean and standard deviation) of the personal or individual characteristics data set (DS 1) for the five base classifiers in isolation and after applying in them the resampling, ensemble (Classic, Boosting-based, Bagging-based and Class-balanced ensembles) and cost-sensitive learning techniques selected | | | | | | | | | | |
| --- | --- | --- | --- | --- | --- | --- | --- | --- | --- | --- |
| **Technique** | **Base classifiers** | | | | | | | | | |
|  | **C4.5** | | **ADTree** | | **SMO** | | **KNN** | | **RF** | |
|  | **AUC** | | **AUC** | | **AUC** | | **AUC** | | **AUC** | |
| None | 0.475 | ±0.091 | 0.579 | ±0.117 | 0.494 | ±0.016 | 0.504 | ±0.126 | 0.492 | ±0.111 |
|  | Resampling Techniques | | | | | | | | | |
| SMOTE | 0.474 | ±0.134 | 0.561 | ±0.123 | 0.488 | ±0.096 | 0.487 | ±0.120 | 0.522 | ±0.113 |
| ROS | 0.454 | ±0.117 | 0.570 | ±0.129 | 0.496 | ±0.100 | 0.488 | ±0.121 | 0.497 | ±0.114 |
| RUS | 0.495 | ±0.103 | 0.565 | ±0.131 | 0.505 | ±0.109 | 0.517 | ±0.129 | 0.490 | ±0.124 |
| ENN | 0.500 | ±0.006 | 0.563 | ±0.128 | 0.491 | ±0.027 | 0.505 | ±0.137 | 0.496 | ±0.121 |
|  | Classic Ensembles | | | | | | | | | |
| ADB1 | 0.435 | ±0.117 | 0.472 | ±0.115 | 0.501 | ±0.101 | 0.476 | ±0.135 | - | - |
| M1 | 0.454 | ±0.113 | 0.475 | ±0.120 | 0.511 | ±0.124 | 0.469 | ±0.109 | - | - |
| BAG | 0.496 | ±0.117 | 0.579 | ±0.109 | 0.512 | ±0.118 | 0.502 | ±0.120 | - | - |
| Decorate | 0.422 | ±0.124 | 0.501 | ±0.120 | 0.494 | ±0.016 | 0.433 | ±0.113 | - | - |
|  | Boosting-based Ensembles | | | | | | | | | |
| SBO | 0.483 | ±0.121 | 0.513 | ±0.122 | 0.509 | ±0.129 | 0.482 | ±0.118 | - | - |
| RUSB | 0.464 | ±0.124 | 0.486 | ±0.114 | 0.485 | ±0.128 | 0.458 | ±0.119 | - | - |
|  | Bagging-based Ensembles | | | | | | | | | |
| OBAG | 0.492 | ±0.112 | 0.573 | ±0.107 | 0.554 | ±0.116 | 0.483 | ±0.111 | - | - |
| UBAG | 0.528 | ±0.119 | 0.579 | ±0.106 | 0.568 | ±0.114 | 0.528 | ±0.119 | - | - |
| SBAG | 0.533 | ±0.112 | 0.583 | ±0.105 | 0.551 | ±0.116 | 0.524 | ±0.112 | - | - |
|  | Cost-sensitive Classification | | | | | | | | | |
| MetaCost | 0.499 | ±0.013 | 0.560 | ±0.117 | 0.485 | ±0.036 | 0.508 | ±0.135 | - | - |
| CS-Classifier | 0.480 | ±0.060 | 0.574 | ±0.122 | 0.474 | ±0.061 | 0.505 | ±0.125 | - | - |
|  | Class-balanced Ensembles with a Cost-sensitive Classifier | | | | | | | | | |
| CS-OBAG | 0.521 | ±0.111 | 0.574 | ±0.107 | 0.564 | ±0.116 | 0.485 | ±0.113 | - | - |
| CS-UBAG | 0.538 | ±0.112 | 0.581 | ±0.108 | 0.578 | ±0.114 | 0.528 | ±0.121 | - | - |
| CS-SBAG | 0.545 | ±0.109 | 0.584 | ±0.104 | 0.551 | ±0.116 | 0.523 | ±0.113 | - | - |
