# Supplementary material for: A Field-Based Approach to Determine Soft Tissue Injury Risk in Elite Futsal Using Novel Machine Learning Techniques
Source: Front Psychol. 2021 Feb 5;12:610210. doi: 10.3389/fpsyg.2021.610210 (PMC7892460; doi:10.3389/fpsyg.2021.610210)
Supplement: Supplementary File 10 — AUC results (mean and standard deviation) of the Athlete Burnout data set (DS 3) for the four base classifiers in isolation and after applying in them the resampling ensemble (Classic, Boosting-based, Bagging-based, and Class-balanced ensembles) and cost-sensitive learning techniques selected. [file Table_10.DOCX]

| **Supplementary file 10.** AUC results (mean and standard deviation) of the Athlete Burnout data set (DS 3) for the four base classifiers in isolation and after applying in them the resampling. ensemble (Classic, Boosting-based, Bagging-based and Class-balanced ensembles) and cost-sensitive learning techniques selected | | | | | | | | | | |
| --- | --- | --- | --- | --- | --- | --- | --- | --- | --- | --- |
| **Technique** | **Base classifiers** | | | | | | | | | |
|  | **C4.5** | | **ADTree** | | **SMO** | | **KNN** | | **RF** | |
|  | **AUC** | | **AUC** | | **AUC** | | **AUC** | | **AUC** | |
| None | 0.500 | ±0.000 | 0.558 | ±0.127 | 0.495 | ±0.024 | 0.642 | ±0.117 | 0.633 | ±0.121 |
|  | Resampling Techniques | | | | | | | | | |
| SMOTE | 0.543 | ±0.122 | 0.537 | ±0.126 | 0.511 | ±0.102 | 0.614 | ±0.114 | 0.598 | ±0.114 |
| ROS | 0.542 | ±0.123 | 0.568 | ±0.121 | 0.532 | ±0.102 | 0.642 | ±0.118 | 0.630 | ±0.120 |
| RUS | 0.494 | ±0.044 | 0.558 | ±0.123 | 0.525 | ±0.097 | 0.604 | ±0.121 | 0.592 | ±0.127 |
| ENN | 0.500 | ±0.000 | 0.553 | ±0.125 | 0.502 | ±0.038 | 0.619 | ±0.127 | 0.618 | ±0.128 |
|  | Classic Ensembles | | | | | | | | | |
| ADB1 | 0.577 | ±0.125 | 0.617 | ±0.126 | 0.523 | ±0.099 | 0.627 | ±0.127 | - | - |
| M1 | 0.564 | ±0.123 | 0.615 | ±0.126 | 0.560 | ±0.122 | 0.630 | ±0.118 | - | - |
| BAG | 0.506 | ±0.106 | 0.579 | ±0.128 | 0.530 | v0.118 | 0.636 | ±0.120 | - | - |
| Decorate | 0.521 | ±0.122 | 0.588 | ±0.133 | 0.495 | ±0.024 | 0.610 | ±0.124 | - | - |
|  | Boosting-based Ensembles | | | | | | | | | |
| SBO | 0.596 | ±0.123 | 0.594 | ±0.126 | 0.570 | ±0.119 | 0.619 | ±0.122 | - | - |
| RUSB | 0.591 | ±0.122 | 0.612 | ±0.126 | 0.572 | ±0.122 | 0.624 | ±0.121 | - | - |
|  | Bagging-based Ensembles | | | | | | | | | |
| OBAG | 0.610 | ±0.124 | 0.583 | ±0.126 | 0.588 | ±0.121 | 0.636 | ±0.120 | - | - |
| UBAG | 0.562 | ±0.133 | 0.577 | ±0.125 | 0.568 | ±0.119 | 0.617 | ±0.123 | - | - |
| SBAG | 0.585 | ±0.124 | 0.581 | ±0.126 | 0.570 | ±0.119 | 0.622 | ±0.116 | - | - |
|  | Cost-sensitive Classification | | | | | | | | | |
| MetaCost | 0.500 | ±0.000 | 0.555 | ±0.125 | 0.512 | ±0.048 | 0.562 | ±0.138 | - | - |
| CS-Classifier | 0.500 | ±0.000 | 0.562 | ±0.125 | 0.523 | ±0.063 | 0.643 | ±0.118 | - | - |
|  | Class-balanced Ensembles with a Cost-sensitive Classifier | | | | | | | | | |
| CS-OBAG | 0.592 | ±0.128 | 0.581 | ±0.128 | 0.580 | ±0.122 | 0.635 | ±0.119 | - | - |
| CS-UBAG | 0.564 | ±0.122 | 0.578 | ±0.127 | 0.568 | ±0.124 | 0.616 | ±0.125 | - | - |
| CS-SBAG | 0.583 | ±0.119 | 0.579 | ±0.127 | 0.565 | ±0.121 | 0.624 | ±0.116 | - | - |
