# Supplementary material for: A Field-Based Approach to Determine Soft Tissue Injury Risk in Elite Futsal Using Novel Machine Learning Techniques
Source: Front Psychol. 2021 Feb 5;12:610210. doi: 10.3389/fpsyg.2021.610210 (PMC7892460; doi:10.3389/fpsyg.2021.610210)
Supplement: Supplementary File 12 — AUC results (mean and standard deviation) of the self-perceived chronic ankle instability data set (DS 5) for the four base classifiers in isolation and after applying in them the resampling. ensemble (Classic, Boosting-based, Bagging-based, and Class-balanced ensembles) and cost-sensitive learning techniques selected. [file Table_12.DOCX]

| **Supplementary file 12.** AUC results (mean and standard deviation) of the self-perceived chronic ankle instability data set (DS 5) for the four base classifiers in isolation and after applying in them the resampling. ensemble (Classic, Boosting-based, Bagging-based and Class-balanced ensembles) and cost-sensitive learning techniques selected | | | | | | | | | | |
| --- | --- | --- | --- | --- | --- | --- | --- | --- | --- | --- |
| **Technique** | **Base classifiers** | | | | | | | | | |
|  | **C4.5** | | **ADTree** | | **SMO** | | **KNN** | | **RF** | |
|  | **AUC** | | **AUC** | | **AUC** | | **AUC** | | **AUC** | |
| None | 0.500 | ±0.000 | 0.596 | ±0.108 | 0.497 | ±0.014 | 0.596 | ±0.109 | 0.598 | ±0.111 |
|  | Resampling Techniques | | | | | | | | | |
| SMOTE | 0.572 | ±0.108 | 0.564 | ±0.107 | 0.520 | ±0.085 | 0.552 | ±0.108 | 0.556 | ±0.108 |
| ROS | 0.551 | ±0.100 | 0.597 | ±0.115 | 0.532 | ±0.079 | 0.592 | ±0.118 | 0.596 | ±0.118 |
| RUS | 0.517 | ±0.075 | 0.582 | ±0.118 | 0.530 | ±0.087 | 0.582 | ±0.120 | 0.588 | ±0.122 |
| ENN | 0.500 | ±0.000 | 0.590 | ±0.116 | 0.500 | ±0.019 | 0.589 | ±0.120 | 0.589 | ±0.120 |
|  | Classic Ensembles | | | | | | | | | |
| ADB1 | 0.595 | ±0.108 | 0.597 | ±0.109 | 0.526 | ±0.091 | 0.596 | ±0.110 | - | - |
| M1 | 0.599 | ±0.113 | 0.595 | ±0.109 | 0.605 | ±0.115 | 0.595 | ±0.108 | - | - |
| BAG | 0.583 | ±0.111 | 0.600 | ±0.112 | 0.543 | ±0.085 | 0.597 | ±0.112 | - | - |
| Decorate | 0.519 | ±0.122 | 0.508 | ±0.117 | 0.497 | ±0.014 | 0.509 | ±0.118 | - | - |
|  | Boosting-based Ensembles | | | | | | | | | |
| SBO | 0.558 | ±0.114 | 0.551 | ±0.112 | 0.559 | ±0.116 | 0.541 | ±0.110 | - | - |
| RUSB | 0.584 | ±0.111 | 0.593 | ±0.113 | 0.579 | ±0.123 | 0.590 | ±0.114 | - | - |
|  | Bagging-based Ensembles | | | | | | | | | |
| OBAG | 0.588 | ±0.116 | 0.604 | ±0.114 | 0.604 | ±0.111 | 0.597 | ±0.115 | - | - |
| UBAG | 0.612 | ±0.118 | 0.599 | ±0.113 | 0.595 | ±0.123 | 0.594 | ±0.112 | - | - |
| SBAG | 0.567 | ±0.113 | 0.576 | ±0.113 | 0.606 | ±0.116 | 0.566 | ±0.115 | - | - |
|  | Cost-sensitive Classification | | | | | | | | | |
| MetaCost | 0.499 | ±0.007 | 0.518 | ±0.123 | 0.498 | ±0.024 | 0.478 | ±0.126 | - | - |
| CS-Classifier | 0.501 | ±0.030 | 0.596 | ±0.109 | 0.532 | ±0.054 | 0.596 | ±0.110 | - | - |
|  | Class-balanced Ensembles with a Cost-sensitive Classifier | | | | | | | | | |
| CS-OBAG | 0.589 | ±0.116 | 0.604 | ±0.113 | 0.604 | ±0.113 | 0.597 | ±0.115 | - | - |
| CS-UBAG | 0.608 | ±0.117 | 0.601 | ±0.113 | 0.599 | ±0.113 | 0.594 | ±0.114 | - | - |
| CS-SBAG | 0.555 | ±0.111 | 0.574 | ±0.113 | 0.602 | ±0.112 | 0.556 | ±0.113 | - | - |
