# Supplementary material for: A Field-Based Approach to Determine Soft Tissue Injury Risk in Elite Futsal Using Novel Machine Learning Techniques
Source: Front Psychol. 2021 Feb 5;12:610210. doi: 10.3389/fpsyg.2021.610210 (PMC7892460; doi:10.3389/fpsyg.2021.610210)
Supplement: Supplementary File 13 — AUC results (mean and standard deviation) of the lower extremity joint ranges of motion data set (DS 6) for the five base classifiers in isolation and after applying in them the resampling, ensemble (Classic, Boosting-based, Bagging-based, and Class-balanced ensembles) and cost-sensitive learning techniques selected. [file Table_13.DOCX]

| **Supplementary file 13.** AUC results (mean and standard deviation) of the lower extremity joint ranges of motion data set (DS 6) for the five base classifiers in isolation and after applying in them the resampling, ensemble and cost-sensitive learning techniques selected | | | | | | | | | | | |  |
| --- | --- | --- | --- | --- | --- | --- | --- | --- | --- | --- | --- | --- |
| **Technique** | **Base classifiers** | | | | | | | | |  |  | |
|  | **C4.5** | | **ADTree** | | **SMO** | | **KNN** | | **RF** | | | |
|  | **AUC** | | **AUC** | | **AUC** | | **AUC** | | **AUC** | | | |
| None | 0.629 | ±0.115 | **0.754** | **±0.122** | 0.567 | ±0.098 | 0.591 | ±0.125 | 0.690 | | ±0.125 | |
|  | Resampling Techniques | | | | | | | | | | |  |
| SMOTE | 0.614 | ±0.121 | **0.710** | **±0.126** | 0.563 | ±0.101 | 0.601 | ±0.117 | 0.679 | | ±0.117 | |
| ROS | 0.620 | ±0.115 | **0.745** | **±0.126** | 0.567 | ±0.097 | 0.592 | ±0.120 | **0.710** | | **±0.111** | |
| RUS | 0.640 | ±0.122 | 0.692 | ±0.130 | 0.595 | ±0.117 | 0.624 | ±0.122 | 0.688 | | ±0.121 | |
| ENN | 0.602 | ±0.113 | 0.695 | ±0.130 | 0.561 | ±0.102 | 0.601 | ±0.126 | 0.674 | | ±0.125 | |
|  | Classic Ensembles | | | | | | | | | | |  |
| ADB1 | 0.602 | ±0.088 | **0.750** | **±0.112** | 0.575 | ±0.099 | 0.530 | ±0.121 | - | | - | |
| M1 | 0.614 | ±0.092 | **0.726** | **±0.121** | 0.575 | ±0.099 | 0.556 | ±0.115 | - | | - | |
| BAG | **0.742** | **±0.105** | **0.755** | **±0.110** | 0.677 | ±0.111 | 0.609 | ±0.115 | - | | - | |
| Decorate | 0.681 | ±0.125 | **0.738** | **±0.113** | 0.569 | ±0.098 | 0.609 | ±0.124 | - | | - | |
|  | Boosting-based Ensembles | | | | | | | | | | |  |
| SBO | 0.652 | ±0.113 | 0.669 | ±0.129 | 0.573 | ±0.098 | 0.577 | ±0.143 | - | | - | |
| RUSB | 0.672 | ±0.113 | 0.675 | ±0.128 | 0.616 | ±0.104 | 0.628 | ±0.126 | - | | - | |
|  | Bagging-based Ensembles | | | | | | | | | | |  |
| OBAG | **0.758** | **±0.088** | **0.755** | **±0.109** | 0.677 | ±0.110 | 0.611 | ±0.114 | - | | - | |
| UBAG | **0.758** | **±0.088** | **0.735** | **±0.107** | 0.685 | ±0.107 | 0.652 | ±0.108 | - | | - | |
| SBAG | **0.736** | **±0.092** | **0.735** | **±0.106** | 0.681 | ±0.110 | 0.630 | ±0.116 | - | | - | |
|  | Cost-sensitive Classification | | | | | | | | | | |  |
| MetaCost | 0.620 | ±0.115 | **0.728** | **±0.125** | 0.564 | ±0.096 | 0.605 | ±0.129 | - | | - | |
| CS-Classifier | 0.641 | ±0.112 | **0.757** | **±0.124** | 0.567 | ±0.098 | 0.500 | ±0.000 | - | | - | |
|  | Class-balanced Ensembles with a Cost-sensitive Classifier | | | | | | | | | | |  |
| CS-OBAG | **0.746** | **±0.083** | **0.755** | **±0.108** | 0.677 | ±0.111 | 0.607 | ±0.113 | - | | - | |
| CS-UBAG | **0.755** | **±0.086** | **0.737** | **±0.106** | 0.686 | ±0.113 | 0.643 | ±0.114 | - | | - | |
| CS-SBAG | **0.733** | **±0.089** | **0.735** | **±0.107** | 0.681 | ±0.110 | 0.629 | ±0.116 | - | | - | |
| In bold are highlighted those learning techniques that built prediction models with AUC scores >0.7. | | | | | | | | | | | |  |
