# Supplementary material for: A Field-Based Approach to Determine Soft Tissue Injury Risk in Elite Futsal Using Novel Machine Learning Techniques
Source: Front Psychol. 2021 Feb 5;12:610210. doi: 10.3389/fpsyg.2021.610210 (PMC7892460; doi:10.3389/fpsyg.2021.610210)
Supplement: Supplementary File 15 — AUC results (mean and standard deviation) of the dynamic postural control data set (DS 6) for the five base classifiers in isolation and after applying in them the resampling, ensemble (Classic, Boosting-based, Bagging-based, and Class-balanced ensembles), and cost-sensitive learning techniques selected. [file Table_15.DOCX]

| **Supplementary file 15.** AUC results (mean and standard deviation) of the dynamic postural control data set (DS 6) for the five base classifiers in isolation and after applying in them the resampling, ensemble and cost-sensitive learning techniques selected | | | | | | | | | | | |
| --- | --- | --- | --- | --- | --- | --- | --- | --- | --- | --- | --- |
| **Technique** | **Base classifiers** | | | | | | | | | | |
|  | **C4.5** | | **ADTree** | | **SMO** | | **KNN** | | | **RF** | |
|  | **AUC** | | **AUC** | | **AUC** | | **AUC** | | | **AUC** | |
| None | 0.606 | ±0.127 | 0.644 | ±0.119 | 0.527 | ±0.091 | 0.587 | ±0.132 | 0.564 | | ±0.133 |
|  | Resampling Techniques | | | | | | | | | | |
| SMOTE | 0.634 | ±0.129 | 0.652 | ±0.115 | 0.623 | ±0.115 | 0.590 | ±0.138 | 0.571 | | ±0.142 |
| ROS | 0.590 | ±0.123 | 0.640 | ±0.119 | 0.607 | ±0.117 | 0.564 | ±0.132 | 0.560 | | ±0.141 |
| RUS | 0.619 | ±0.130 | 0.623 | ±0.127 | 0.601 | ±0.124 | 0.602 | ±0.136 | 0.610 | | ±0.134 |
| ENN | - | - | 0.638 | ±0.128 | 0.533 | ±0.097 | 0.579 | ±0.143 | 0.575 | | ±0.138 |
|  | Classic Ensembles | | | | | | | | | | |
| ADB1 | 0.618 | ±0.125 | 0.609 | ±0.130 | 0.578 | ±0.121 | 0.544 | ±0.127 | - | | - |
| M1 | 0.633 | ±0.125 | 0.674 | ±0.130 | 0.606 | ±0.121 | 0.564 | ±0.124 | - | | - |
| BAG | 0.624 | ±0.123 | 0.675 | ±0.118 | 0.582 | ±0.127 | 0.591 | ±0.135 | - | | - |
| Decorate | 0.508 | ±0.132 | 0.616 | ±0.133 | 0.518 | ±0.079 | 0.521 | ±0.139 | - | | - |
|  | Boosting-based Ensembles | | | | | | | | | | |
| SBO | 0.580 | ±0.135 | 0.574 | ±0.160 | 0.662 | ±0.139 | 0.571 | ±0.136 | - | | - |
| RUSB | 0.594 | ±0.125 | 0.605 | ±0.132 | 0.600 | ±0.134 | 0.591 | ±0.136 | - | | - |
|  | Bagging-based Ensembles | | | | | | | | | | |
| OBAG | 0.642 | ±0.124 | 0.674 | ±0.122 | 0.630 | ±0.128 | 0.586 | ±0.134 | - | | - |
| UBAG | 0.677 | ±0.115 | 0.677 | ±0.119 | 0.641 | ±0.129 | 0.619 | ±0.137 | - | | - |
| SBAG | 0.641 | ±0.133 | 0.671 | ±0.120 | 0.628 | ±0.131 | 0.592 | ±0.140 | - | | - |
|  | Cost-sensitive Classification | | | | | | | | | | |
| MetaCost | 0.569 | ±0.113 | 0.659 | ±0.122 | 0.541 | ±0.101 | 0.585 | ±0.146 | - | | - |
| CS-Classifier | 0.592 | ±0.126 | 0.644 | ±0.117 | 0.540 | ±0.105 | 0.591 | ±0.134 | - | | - |
|  | Class-balanced Ensembles with a Cost-sensitive Classifier | | | | | | | | | | |
| CS-OBAG | 0.663 | ±0.125 | 0.674 | ±0.120 | 0.647 | ±0.131 | 0.582 | ±0.134 | - | | - |
| CS-UBAG | **0.701** | **±0.114** | 0.680 | ±0.117 | 0.657 | ±0.128 | 0.605 | ±0.139 | - | | - |
| CS-SBAG | 0.663 | ±0.130 | 0.674 | ±0.120 | 0.638 | ±0.130 | 0.592 | ±0.138 | - | | - |
| In bold are highlighted those learning techniques that built prediction models with AUC scores >0.7 | | | | | | | | | | | |

| **Supplementary file 16.** AUC results (mean and standard deviation) of the measures obtained through questionnaires data set (DS 6) for the five base classifiers in isolation and after applying in them the resampling, ensemble (Classic, Boosting-based, Bagging-based and Class-balanced ensembles) and cost-sensitive learning techniques selected. | | | | | | | | | | |
| --- | --- | --- | --- | --- | --- | --- | --- | --- | --- | --- |
| **Technique** | **Base classifiers** | | | | | | | | | |
|  | **C4.5** | | **ADTree** | | **SMO** | | **KNN** | | **RF** | |
|  | **AUC** | | **AUC** | | **AUC** | | **AUC** | | **AUC** | |
| None | 0.460 | ±0.089 | 0.506 | ±0.133 | 0.518 | ±0.096 | 0.496 | ±0.136 | 0.443 | ±0.131 |
|  | Resampling Techniques | | | | | | | | | |
| SMOTE | 0.508 | ±0.137 | 0.528 | ±0.137 | 0.517 | ±0.100 | 0.458 | ±0.130 | 0.445 | ±0.135 |
| ROS | 0.451 | ±0.113 | 0.510 | ±0.133 | 0.527 | ±0.100 | 0.485 | ±0.134 | 0.446 | ±0.124 |
| RUS | 0.480 | ±0.125 | 0.515 | ±0.135 | 0.527 | ±0.125 | 0.517 | ±0.139 | 0.469 | ±0.131 |
| ENN | 0.474 | ±0.093 | 0.505 | ±0.131 | 0.518 | ±0.102 | 0.498 | ±0.140 | 0.467 | ±0.131 |
|  | Classic Ensembles | | | | | | | | | |
| ADB1 | **-** | **-** | 0.505 | ±0.105 | 0.524 | ±0.113 | 0.489 | ±0.126 | - | - |
| M1 | 0.479 | ±0.091 | 0.497 | ±0.107 | 0.527 | ±0.111 | 0.483 | ±0.121 | - | - |
| BAG | 0.489 | ±0.128 | 0.515 | ±0.130 | 0.548 | ±0.133 | 0.502 | ±0.133 | - | - |
| Decorate | 0.468 | ±0.135 | 0.494 | ±0.138 | 0.530 | ±0.099 | 0.455 | ±0.138 | - | - |
|  | Boosting-based Ensembles | | | | | | | | | |
| SBO | 0.504 | ±0.112 | 0.506 | ±0.122 | - | - | 0.470 | ±0.139 | - | - |
| RUSB | 0.495 | ±0.115 | 0.508 | ±0.104 | 0.530 | ±0.127 | 0.518 | ±0.134 | - | - |
|  | Bagging-based Ensembles | | | | | | | | | |
| OBAG | 0.468 | ±0.126 | 0.516 | ±0.129 | 0.549 | ±0.133 | 0.490 | ±0.130 | - | - |
| UBAG | 0.509 | ±0.134 | 0.529 | ±0.128 | 0.558 | ±0.136 | 0.519 | ±0.133 | - | - |
| SBAG | 0.537 | ±0.124 | 0.532 | ±0.128 | 0.544 | ±0.133 | 0.498 | ±0.134 | - | - |
|  | Cost-sensitive Classification | | | | | | | | | |
| MetaCost | 0.466 | ±0.087 | 0.500 | ±0.128 | 0.533 | ±0.105 | 0.478 | ±0.129 | - | - |
| CS-Classifier | 0.450 | ±0.102 | 0.507 | ±0.130 | 0.530 | ±0.102 | 0.496 | ±0.138 | - | - |
|  | Class-balanced Ensembles with a Cost-sensitive Classifier | | | | | | | | | |
| CS-OBAG | 0.477 | ±0.125 | 0.518 | ±0.128 | 0.550 | ±0.135 | 0.486 | ±0.132 | - | - |
| CS-UBAG | 0.515 | ±0.127 | 0.530 | ±0.131 | 0.556 | ±0.137 | 0.516 | ±0.135 | - | - |
| CS-SBAG | 0.537 | ±0.123 | 0.532 | ±0.128 | 0.548 | ±0.133 | 0.499 | ±0.135 | - | - |

| **Supplementary file 17.** AUC results (mean and standard deviation) of the field-based tests of neuromuscular performance data set (DS 6) for the five base classifiers in isolation and after applying in them the resampling, ensemble and cost-sensitive learning techniques selected | | | | | | | | | | |
| --- | --- | --- | --- | --- | --- | --- | --- | --- | --- | --- |
| **Technique** | **Base classifiers** | | | | | | | | | |
|  | **C4.5** | | **ADTree** | | **SMO** | | **KNN** | | **RF** | |
|  | **AUC** | | **AUC** | | **AUC** | | **AUC** | | **AUC** | |
| None | 0.598 | ±0.097 | **0.758** | **±0.084** | 0.563 | ±0.075 | **0.747** | **±0.098** | **0.742** | **±0.100** |
|  | Resampling Techniques | | | | | | | | | |
| SMOTE | **0.718** | **±0.105** | **0.753** | **±0.088** | 0.685 | ±0.112 | **0.740** | **±0.101** | **0.737** | **±0.105** |
| ROS | **0.704** | **±0.110** | **0.760** | **±0.090** | 0.685 | ±0.126 | **0.749** | **±0.101** | **0.745** | **±0.100** |
| RUS | 0.679 | ±0.118 | **0.749** | **±0.093** | 0.675 | ±0.124 | **0.745** | **±0.100** | **0.742** | **±0.105** |
| ENN | 0.584 | ±0.098 | **0.756** | **±0.091** | 0.559 | ±0.075 | **0.747** | **±0.102** | **0.738** | **±0.105** |
|  | Classic Ensembles | | | | | | | | | |
| ADB1 | **0.756** | **±0.094** | **0.763** | **±0.086** | **0.776** | **±0.088** | **0.738** | **±0.101** | - | - |
| M1 | **0.759** | **±0.086** | **0.751** | **±0.093** | **0.757** | **±0.091** | **0.748** | **±0.101** | - | - |
| BAG | **0.727** | **±0.088** | **0.763** | **±0.087** | 0.661 | ±0.127 | **0.756** | **±0.094** | - | - |
| Decorate | **0.710** | **±0.102** | **0.732** | **±0.095** | 0.564 | ±0.075 | **0.708** | **±0.108** | - | - |
|  | Boosting-based Ensembles | | | | | | | | | |
| SBO | **0.739** | **±0.104** | **0.747** | **±0.104** | **0.749** | **±0.102** | **0.735** | **±0.102** | - | - |
| RUSB | **0.751** | **±0.091** | **0.759** | **±0.089** | **0.758** | **±0.089** | **0.745** | **±0.097** | - | - |
|  | Bagging-based Ensembles | | | | | | | | | |
| OBAG | **0.753** | **±0.089** | **0.766** | **±0.087** | **0.750** | **±0.099** | **0.759** | **±0.096** | - | - |
| UBAG | **0.747** | **±0.084** | **0.755** | **±0.087** | **0.752** | **±0.094** | **0.758** | **±0.092** | - | - |
| SBAG | **0.769** | **±0.099** | **0.776** | **±0.092** | **0.771** | **±0.101** | **0.769** | **±0.100** | - | - |
|  | Cost-sensitive Classification | | | | | | | | | |
| MetaCost | 0.539 | ±0.081 | **0.724** | **±0.110** | 0.500 | ±0.000 | 0.519 | ±0.200 | - | - |
| CS-Classifier | 0.641 | ±0.112 | **0.756** | **±0.087** | 0.500 | ±0.000 | **0.751** | **±0.099** | - | - |
|  | Class-balanced Ensembles with a Cost-sensitive Classifier | | | | | | | | | |
| CS-OBAG | **0.759** | **±0.095** | **0.767** | **±0.088** | **0.760** | **±0.103** | **0.763** | **±0.097** | - | - |
| CS-UBAG | **0.748** | **±0.089** | **0.757** | **±0.088** | **0.767** | **±0.096** | **0.761** | **±0.095** | - | - |
| CS-SBAG | **0.770** | **±0.104** | **0.776** | **±0.092** | **0.768** | **±0.100** | **0.772** | **±0.101** | - | - |
| In bold are highlighted those learning techniques that built prediction models with AUC scores >0.7. | | | | | | | | | | |

| **Supplementary file 18.** AUC results (mean and standard deviation) of the global data set (DS 11) for the five base classifiers in isolation and after applying in them the resampling, ensemble (Classic, Boosting-based, Bagging-based and Class-balanced ensembles) and cost-sensitive learning techniques selected | | | | | | | | | | |
| --- | --- | --- | --- | --- | --- | --- | --- | --- | --- | --- |
| **Technique** | **Base classifiers** | | | | | | | | | |
|  | **C4.5** | | **ADTree** | | **SMO** | | **KNN** | | **RF** | |
|  | **AUC** | | **AUC** | | **AUC** | | **AUC** | | **AUC** | |
| None | 0.642 | ±0.124 | **0.741** | **±0.119** | 0.568 | ±0.086 | **0.704** | **±0.131** | **0.713** | **±0.135** |
|  | Resampling Techniques | | | | | | | | | |
| SMOTE | **0.709** | **±0.130** | **0.738** | **±0.121** | 0.651 | ±0.128 | **0.700** | **±0.129** | **0.711** | **±0.139** |
| ROS | 0.694 | ±0.130 | **0.738** | **±0.122** | 0.659 | ±0.127 | **0.704** | **±0.131** | **0.712** | **±0.136** |
| RUS | 0.663 | ±0.131 | **0.720** | **±0.126** | 0.645 | ±0.129 | 0.698 | ±0.120 | **0.708** | **±0.137** |
| ENN | 0.637 | ±0.123 | **0.731** | **±0.124** | 0.567 | ±0.093 | 0.697 | ±0.130 | **0.707** | **±0.136** |
|  | Classic Ensembles | | | | | | | | | |
| ADB1 | **0.746** | **±0.124** | **0.769** | **±0.131** | **0.722** | **±0.138** | 0.691 | ±0.135 | - | - |
| M1 | **0.754** | **±0.110** | **0.742** | **±0.144** | **0.797** | **±0.131** | 0.690 | ±0.136 | - | - |
| BAG | **0.740** | **±0.115** | **0.743** | **±0.116** | 0.694 | ±0.131 | **0.716** | **±0.127** | - | - |
| Decorate | **0.709** | **±0.127** | **0.720** | **±0.124** | 0.569 | ±0.087 | 0.676 | ±0.141 | - | - |
|  | Boosting-based Ensembles | | | | | | | | | |
| SBO | **0.715** | **±0.138** | **0.749** | **±0.061** | **0.740** | **±0.102** | **0.707** | **±0.132** | - | - |
| RUSB | **0.736** | **±0.121** | **0.748** | **±0.138** | **0.752** | **±0.118** | **0.710** | **±0.128** | - | - |
|  | Bagging-based Ensembles | | | | | | | | | |
| OBAG | **0.744** | **±0.112** | **0.741** | **±0.116** | **0.742** | **±0.125** | **0.720** | **±0.126** | - | - |
| UBAG | **0.742** | **±0.111** | **0.739** | **±0.119** | **0.737** | **±0.121** | **0.719** | **±0.120** | - | - |
| SBAG | **0.751** | **±0.118** | **0.745** | **±0.119** | **0.750** | **±0.124** | **0.724** | **±0.125** | - | - |
|  | Cost-sensitive Classification | | | | | | | | | |
| MetaCost | 0.572 | ±0.120 | 0.698 | ±0.134 | 0.500 | ±0.000 | 0.604 | ±0.147 | - | - |
| CS-Classifier | 0.685 | ±0.129 | **0.739** | **±0.124** | 0.500 | ±0.000 | **0.706** | **±0.128** | - | - |
|  | Class-balanced Ensembles with a Cost-sensitive Classifier | | | | | | | | | |
| CS-OBAG | **0.751** | **±0.107** | **0.742** | **±0.115** | **0.747** | **±0.121** | **0.715** | **±0.126** | - | - |
| CS-UBAG | **0.749** | **±0.105** | **0.741** | **±0.119** | **0.747** | **±0.116** | **0.722** | **±0.124** | - | - |
| CS-SBAG | **0.755** | **±0.115** | **0.746** | **±0.119** | **0.750** | **±0.121** | **0.719** | **±0.127** | - | - |
| In bold are highlighted those learning techniques that built prediction models with AUC scores >0.7. | | | | | | | | | | |

| **Supplementary file 19: schemes of the algorithms selected in data sets (DS) 6, 8, 10 and 11** |
| --- |
| **Lower extremity joint ranges of motion (DS – 6)** |
| CS-Classifier [ADTree] |
| weka.classifiers.meta.MultiSearch -E FM -search "weka.core.setupgenerator.MathParameter -property classifier.numOfBoostingIterations -min 5.0 -max 50.0 -step 1.0 -base 10.0 -expression I" -class-label 1 -algorithm "weka.classifiers.meta.multisearch.DefaultSearch -sample-size 100.0 -initial-folds 2 -subsequent-folds 10 -initial-test-set . -subsequent-test-set . -num-slots 1" -log-file /Applications/weka-3-8-3 -S 1 -W weka.classifiers.meta.CostSensitiveClassifier -- -cost-matrix "[0.0 2.0; 1.0 0.0]" -S 1 -W weka.classifiers.trees.ADTree -- -B 10 -E -3 -S 1 |
| **Dynamic postural control (DS – 8)** |
| CS-UBAG [C4.5] |
| weka.classifiers.meta.MultiSearch -E FM -search "weka.core.setupgenerator.MathParameter -property classifier.classifier.classifier.confidenceFactor -min 0.05 -max 0.75 -step 0.05 -base 10.0 -expression I" -class-label 1 -algorithm "weka.classifiers.meta.multisearch.DefaultSearch -sample-size 100.0 -initial-folds 2 -subsequent-folds 10 -initial-test-set . -subsequent-test-set . -num-slots 1" -log-file /Applications/weka-3-8-3 -S 1 -W weka.classifiers.meta.Bagging -- -P 100 -S 1 -num-slots 1 -I 100 -W weka.classifiers.meta.FilteredClassifier -- -F "weka.filters.supervised.instance.RUS -P 60.0" -S 1 -W weka.classifiers.meta.CostSensitiveClassifier -- -cost-matrix "[0.0 2.0; 1.0 0.0]" -S 1 -W weka.classifiers.trees.J48 -- -C 0.25 -M 2 |
| **Neuromuscular measures from field-based tests (DS – 10)** |
| CS-UBAG [SMO] |
| weka.classifiers.meta.AttributeSelectedClassifier -E "weka.attributeSelection.CfsSubsetEval -P 1 -E 1" -S "weka.attributeSelection.GreedyStepwise -B -T -1.7976931348623157E308 -N -1 -num-slots 1" -W weka.classifiers.meta.MultiSearch -- -E AUC -search "weka.core.setupgenerator.MathParameter -property classifier.classifier.classifier.calibrator.ridge -min -10.0 -max 5.0 -step 1.0 -base 10.0 -expression pow(BASE,I)" -class-label 1 -algorithm "weka.classifiers.meta.multisearch.DefaultSearch -sample-size 100.0 -initial-folds 2 -subsequent-folds 10 -initial-test-set . -subsequent-test-set . -num-slots 1" -log-file /Applications/weka-3-8-3 -S 1 -W weka.classifiers.meta.Bagging -- -P 100 -S 1 -num-slots 1 -I 100 -W weka.classifiers.meta.FilteredClassifier -- -F "weka.filters.supervised.instance.RUS -P 60.0" -S 1 -W weka.classifiers.meta.CostSensitiveClassifier -- -cost-matrix "[0.0 2.0; 1.0 0.0]" -S 1 -W weka.classifiers.functions.SMO -- -C 1.0 -L 0.001 -P 1.0E-12 -N 0 -V -1 -W 1 -K "weka.classifiers.functions.supportVector.PolyKernel -E 1.0 -C 250007" -calibrator "weka.classifiers.functions.Logistic -R 1.0E-8 -M -1 -num-decimal-places 4" |
| **Global (DS – 11)** |
| CS-UBAG [C4.5] |
| weka.classifiers.meta.AttributeSelectedClassifier -E "weka.attributeSelection.CfsSubsetEval -P 1 -E 1" -S "weka.attributeSelection.GreedyStepwise -B -T -1.7976931348623157E308 -N -1 -num-slots 1" -W weka.classifiers.meta.MultiSearch -E FM -search "weka.core.setupgenerator.MathParameter -property classifier.classifier.classifier.confidenceFactor -min 0.05 -max 0.75 -step 0.05 -base 10.0 -expression I" -class-label 1 -algorithm "weka.classifiers.meta.multisearch.DefaultSearch -sample-size 100.0 -initial-folds 2 -subsequent-folds 10 -initial-test-set . -subsequent-test-set . -num-slots 1" -log-file /Applications/weka-3-8-3 -S 1 -W weka.classifiers.meta.Bagging -- -P 100 -S 1 -num-slots 1 -I 100 -W weka.classifiers.meta.FilteredClassifier -- -F "weka.filters.supervised.instance.RUS -P 60.0" -S 1 -W weka.classifiers.meta.CostSensitiveClassifier -- -cost-matrix "[0.0 2.0; 1.0 0.0]" -S 1 -W weka.classifiers.trees.J48 -- -C 0.25 -M 2 |
